# A Novel Analytical Strategy to Detect Specific Markers of Nitrofurazone from Protein-Bound Metabolites in Food Animals

**DOI:** 10.1021/acs.jafc.6c04435

**Published:** 2026-09-18

**Authors:** Sedigheh Barzegar, Bryn O. Shurmer, Christopher O. Miles, Anas El-Aneed, Randy W. Purves

**Affiliations:** † College of Pharmacy and Nutrition, 7235 University of Saskatchewan, Saskatoon, Saskatchewan S7N 5E5, Canada; ‡ Centre for Veterinary Drug Residues, 5737 Canadian Food Inspection Agency, Saskatoon, Saskatchewan S7N 2R3, Canada; § Biotoxin Metrology, 6356 National Research Council, 1411 Oxford Street, Halifax, Nova Scotia B3H 3Z1, Canada; ∥ Norwegian Veterinary Institute, Elizabeth Stephansens vei 1, 1433 Ås, Norway

**Keywords:** nitrofuran, semicarbazide, high-resolution mass spectrometry (HRMS), food safety, deconjugation, substitution, regulatory, MS^3^

## Abstract

Accurate detection of nitrofuran residues, as an indicator of banned drug use, in animal-derived food products is vital for ensuring food safety. The accepted regulatory approach for nitrofurans produces a residue marker from their protein-bound metabolites, which is nonspecific for nitrofurazone (i.e., semicarbazide (SEM)). Since SEM may originate from non-nitrofurazone sources, it is therefore not conclusive for nitrofurazone. Our main objective was to apply novel deconjugation/substitution reactions, which have been used to reverse protein binding, to identify a specific nitrofurazone marker. Bound nitrofurazone metabolites, produced using S9 from bovine liver or homogenized bovine liver tissue, were subjected to basic media and thiol reagents. Analyses carried out using high-resolution mass spectrometry showed that substitution of protein with thiol reagents produced thiol-metabolite conjugates whose structures were supported with stable isotopically labeled experiments. These conjugates can serve as new, specific markers for conclusive detection of nitrofurazone abuse in food animals.

## Graphical abstract

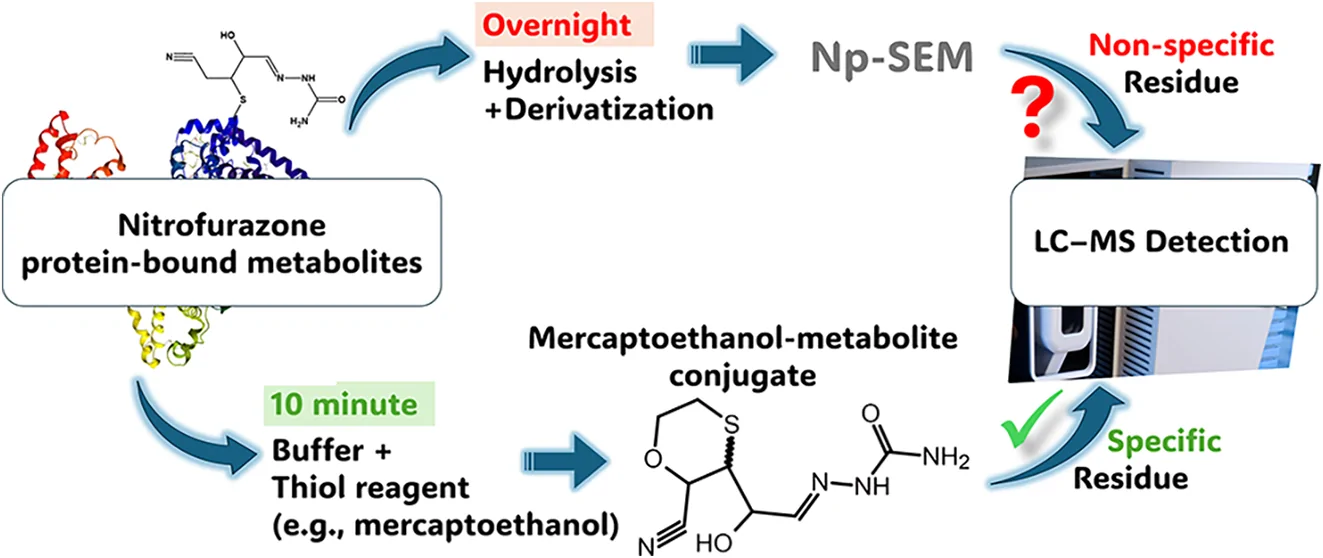

## 1 Introduction

Nitrofurans are a class of antibiotics used to treat gastrointestinal and dermatological disorders, but they can also be used to enhance growth in food animals. They have been widely used due to their cost-effectiveness, accessibility, and efficacy;^1^ however, concerns about the adverse effects of nitrofuran residues (NRs) in animal-derived food products have led to bans in several jurisdictions, including the USA,^2^ Canada,^2^ and the European Union.^3^ The side effects of NRs include allergic reactions, toxicity, and carcinogenic, teratogenic, and mutagenic effects.^1,4–6^ Moreover, extended use of nitrofurans may lead to the emergence of resistant pathogens, which pose significant health risks to humans.^7^ Therefore, detecting NRs in animal-derived food products is crucial to preventing their consumption. However, detecting nitrofurazone residues, in particular, poses challenges due to issues with the specificity of the residue.

Nitrofurans undergo similar metabolic processes. Scheme 1 shows that nitrofurazone is primarily metabolized into open-chain cyano derivatives through a multistep reduction of the nitro group, starting with the formation of a nitro-anion radical and progressing through nitroso and hydroxylamine intermediates.^8,9^ These open-chain cyano derivatives then bind covalently to cellular proteins in a pH-dependent reversible reaction.^8,10^ For nitrofurazone, a radiolabeled study estimated that ≥70% of the metabolites remain unextractable due to covalent binding to protein in the tissues.^11^ These bound metabolites were shown to persist in the tissues;^11–15^ one study showed that the unextractable metabolites were still measurable after 90 days.^15^

**Scheme 1:**
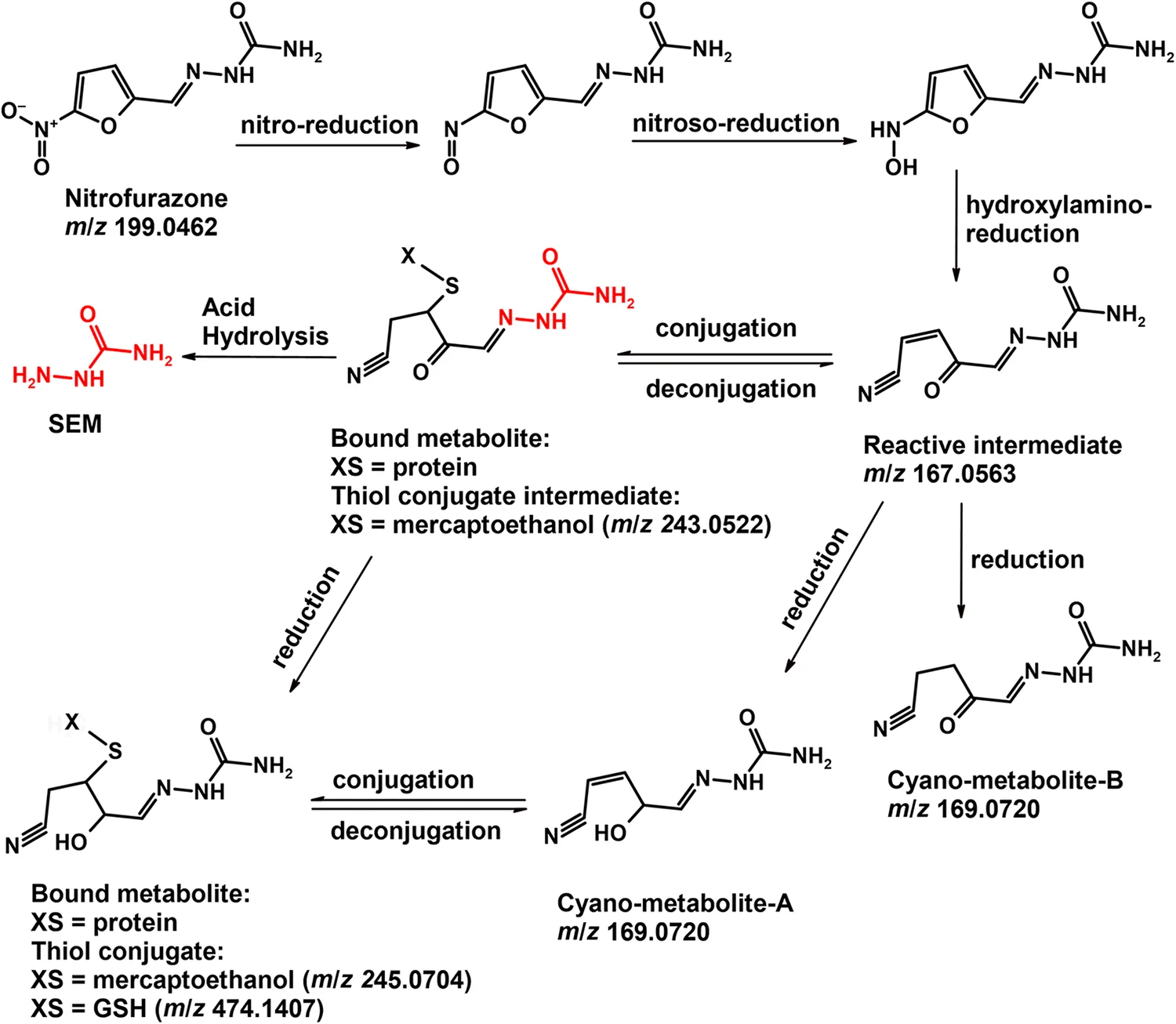
Multistep Nitro-Reduction and Protein/Thiol Binding Pathway Proposed for Nitrofurazone to Show Conjugation/Deconjugation Reactions for Metabolites with Proteins and Thiols^a^

a The red substructure shows the semicarbazide (SEM) portion of the molecule. All *m*/*z* values are exact masses for the [M + H]^+^ ion.

Regulatory methods for nitrofurans rely on cleaving a side chain from the protein-bound metabolites through hydrolysis, followed by derivatization.^2,16–23^ Methods for analysis employ liquid chromatography–tandem mass spectrometry (LC–MS/MS) because of its high specificity and sensitivity.^2,16–23^ For nitrofurazone, shown in Scheme 1, the side chain (the red substructure) is semicarbazide (SEM). Unfortunately, the presence of SEM is not specific to nitrofurazone, and current methods can produce false positives because SEM also originates from non-nitrofurazone sources such as thermal degradation of foamed seals and packaging materials,^24^ decomposition of azodicarbonamide present in flour, canned foods, and disinfection byproducts in dairy products.^25,26^ Additionally, SEM may originate from endogenous/environmental sources (e.g., arginine and creatine) and may be present in some algae, shrimp, eggs,^25^ and honey.^27^ Studies have investigated the hydrolysis step, focusing on optimizing conditions^2,16,17,28,29^ to reduce the time required and improve sensitivity, as well as exploring the use of various derivatizing agents for all NRs.^30,31^ However, these approaches do not address the fundamental lack of SEM specificity for the unambiguous detection of nitrofurazone. Metabolism studies have been carried out to identify the major metabolites of nitrofurazone;^24,32^ however, thus far, these studies have not led to new methods for nitrofurazone detection. Scheme 1 shows that the reactive intermediate can undergo reduction to produce a cyano metabolite as reported previously with the keto group (cyano-metabolite-B)^8,33^ or possibly with a hydroxy group (cyano-metabolite-A).

The goal of our novel approach was to reverse protein binding to release entire metabolites from the protein and then bind the metabolite with a thiol reagent. By doing this, the specific metabolite can be unequivocally linked to nitrofurazone abuse. Previous work with microsomal incubations in the presence of excess glutathione (GSH), followed by the addition of mercaptoethanol, showed the conversion of the GSH-conjugated metabolite (GSH-conjugate) to the mercaptoethanol conjugate. Additionally, microsomal incubations in the presence of excess mercaptoethanol, followed by the addition of microsomal protein, confirmed the conversion of mercaptoethanol conjugates to protein conjugates.^8^ Interactions of these metabolites with either thiol reagents or proteins are reported to be reversible and pH-dependent.^8^ Particularly, compounds with an α,β-unsaturated carbonyl group, similar to nitrofuran cyano-metabolites with an α,β-unsaturated cyanide group, have been reported to conjugate with thiol reagents through a nucleophilic addition reaction^34–36^ known as Michael addition. In a model study, the deconjugation of thiol-bound microcystins, which contain an α,β-unsaturated amide, followed by conjugation with mercaptoethanol, methanethiol, cysteine, and GSH, was demonstrated^36^ in a process known as substitution. For nitrofurans, we hypothesized that either the reactive intermediate and/or the cyano-metabolite-A of nitrofurazone (Scheme 1) can bind to protein through Michael additions; thus, the protein can be substituted with thiol reagents.

This study investigates using this novel strategy with the goal of finding specific nitrofurazone residue(s) from bound metabolite(s) for use in routine screening. To achieve this, ultraperformance liquid chromatography (UPLC) was coupled with; a high-resolution mass spectrometer (i.e., UPLC–HRMS) to identify thiol-metabolite conjugates; a quadrupole linear ion trap (Q–LIT) MS for structural characterization; and a triple quadrupole (QqQ) MS for quantification studies.

## 2 Materials and Methods

### 2.1 Tissue Samples and Bovine Liver S9 Fractions

Federally regulated abattoirs abide by the Safe Food for Canadians Regulations, which define the conditions for humane handling and slaughter of food animals. Tissues from food animals sampled from these establishments are routinely tested for veterinary drugs by the Center for Veterinary Drug Residues at the Canadian Food Inspection Agency (Saskatoon, SK, Canada). Bovine liver tissues that were negative for nitrofurans were repurposed to prepare bovine liver S9 fractions and pseudo-incurred tissue samples, which were stored at −70 and −20 °C, respectively, prior to the studies.

### 2.2 Materials

Nicotinamide-adenine dinucleotide phosphate (NADPH) tetrasodium salt, Tris hydrochloride, and glucose-6-phosphate (G6p) were from Roche Diagnostics GmbH (Mannheim, Germany). Nitrofurazone (≥97.0%, HPLC grade), nitrofurazone-[^13^C,^15^N_2_] (>99.0%, HPLC grade, IUPAC name: (*E*)-2-[(5-nitrofuran-2-yl)­methylene]­hydrazine-1,2-^15^N_2_-1-carboxamide-^13^C), sodium hydroxide, mercaptoethanol (≥99.0%, GC grade), mercaptoethanol-D_4_ (98.0%, CP), potassium chloride, magnesium chloride, sodium carbonate, sodium hydrogen carbonate, and l-glutathione (GSH) (≥98.0%) were from Sigma-Aldrich (St. Louis, MO, USA). Potassium dihydrogen phosphate, disodium hydrogen phosphate, phosphoric acid, formic acid, and acetonitrile (MeCN) were from Fisher Chemical (Ottawa, ON, Canada). Commercial bovine S9 (20 mg/mL, 201­(BOSL191105)) was from Primacyt Cell Culture Technology GmbH (Schwerin, Germany). Ethanol (EtOH) and methanol (MeOH) were obtained from Greenfield Global (Brampton, ON, Canada) and Caledon Laboratory Chemicals (Georgetown, ON, Canada), respectively. The reverse osmosis-purified, deionized, and filtered water was from a Milli-Q Reference water purification system.

### 2.3 *In Vitro* Metabolism

#### 2.3.1 Bovine Liver S9 Fraction Preparation

Bovine liver S9 fractions were prepared according to the procedure used in our previous study.^37^ Briefly, liver tissue was weighed, then homogenized in chilled Tris buffer. Samples were centrifuged (5 °C) at 9000*g* for 20 min and then again at 13,000*g* for 5 min. The supernatant (S9) was stored at −70 °C and was thawed in an ice bath before use. A previous study showed that the in-house liver S9 fractions gave similar results to commercial liver S9 fractions.^37^

#### 2.3.2 Nitrofurazone Incubation Samples and Controls Using Bovine Liver S9 Fractions

The procedure was similar to our previous work.^37^ Incubation samples (with buffer, cofactors, and drug) and incubation controls (drug was added after quenching the metabolism) were prepared for studying nitrofurazone metabolism *in vitro*. For incubation samples, Tris buffer, magnesium chloride, G6p, the bovine liver S9 fraction, and a 1:1 mixture of nitrofurazone and nitrofurazone-[^13^C,^15^N_2_] standard solution in MeOH (final concentration of 40 μg/g for each) were added in a 2 mL Eppendorf tube. Then, NADPH was added to start the incubation.^37^ After incubation in a Thermo Fisher Scientific thermal mixer (version V.490W.001) at 400 rpm and 37 °C for 1 or 4 h, MeCN was added to quench the metabolism. The incubation controls were similar, except that the mixture of nonlabeled and isotopically labeled nitrofurazone was added to the Eppendorf tubes after quenching. All tubes were vortexed and centrifuged at 3000*g* for 5 min at −5 °C. The supernatant was removed and set aside for analysis of free metabolites, whereas the pellet was washed three times with EtOH and three additional times with MeOH. Each time, 500 μL of chilled solvent was added and the pellet was resuspended by vortex mixing, then shaking on a VWR multitube vortexer, Model DVX-2500 (1200 rpm) for 2 min, and centrifugation at 3000*g* for 5 min at −5 °C. The supernatant from the wash steps was kept for analysis, and the pellet was retained for the next steps. The samples and controls were prepared in 4 replicates.

#### 2.3.3 Detection of Nitrofurazone Protein-Bound Metabolites *In Vitro*

##### 2.3.3.1 Protein Substitution Using GSH

A 200 μL aliquot of phosphate buffer (0.1 M, pH 11) was added to the pellet in both sample and control tubes prepared in Section 2.3.2. The tubes were vortex-mixed vigorously for 15 s to disperse the pellet, and then 50 μL of GSH solution (500 μg/g in water) was added. The tubes were shaken in a Thermo Fisher Scientific thermal mixer at 400 rpm and 25 °C for 15 min. After centrifugation (15,000 rpm, 5 min), the supernatant was transferred to amber vials with inserts for UPLC–HRMS analysis.

##### 2.3.3.2 Protein Substitution Using Mercaptoethanol

A 250 μL aliquot of carbonate buffer (0.05 M, pH 9.5) containing 0.5 μL of mercaptoethanol (0.2% mercaptoethanol in carbonate buffer) was added to the pellet in both sample and control tubes prepared in Section 2.3.2. The tube was vortex-mixed, shaken for 10 min, and centrifuged as per Section 2.3.3.1. 200 μL of the supernatant was transferred to an Eppendorf tube. Formic acid (10 μL) was added to change the pH to ≤2, measured with pH paper. It was centrifuged again, and the supernatant was transferred to amber vials with inserts for UPLC–HRMS analysis.

##### 2.3.3.3 Deconjugation of Protein-Bound Nitrofurazone Metabolites

A 250 μL aliquot of carbonate buffer (0.05 M, pH 9.5) was added to the pellet in both sample and control tubes prepared in Section 2.3.2. The tubes were vortex-mixed, shaken for 10 min, and centrifuged as per Section 2.3.3.1. The supernatant was transferred to amber vials with inserts for UPLC–HRMS analysis.

### 2.4 Pseudo-Incurred Metabolites

#### 2.4.1 Pseudo-Incurred Samples Using Bovine Liver Tissue

Bovine liver that was negative for nitrofurans was thawed, and both the tissue and its juice were poured into a grinder (Krups Speedy Pro 720 food processor) and ground several times to produce a fine mash. In a beaker, 20 g of ground tissue was weighed, followed by adding nitrofurazone and nitrofurazone-[^13^C,^15^N_2_] standard solution in MeOH, in several steps and on different spots on top of the mash. This simulated an incurred sample (i.e., pseudo-incurred) containing 2 μg/g each of labeled and nonlabeled drug. Using a spatula to stir for 7 min, the beaker was sealed with parafilm and kept in the fume hood at room temperature or in the incubator at 37 °C for either 1 h or overnight (16 to 18 h) in separate experiments. A portion of fine liver mash prepared the same way, but not spiked, was set aside as a blank. Other concentrations (i.e., 0.05 to 20 μg/g) were prepared in a similar manner while adjusting the nitrofurazone mixture accordingly.

#### 2.4.2 Washing Steps for the Pseudo-Incurred Samples

For the blank, 1.0 g of fine liver mash was used, and for each sample and control, 1.0 g of pseudo-incurred tissue was weighed. To quench the metabolism, 1 mL of MeCN was added to each tube. The tubes were vortex-mixed for 15 s and centrifuged at 3000*g* for 5 min at −5 °C. The supernatant was removed, leaving the entire pellet undisturbed. The supernatant was kept for separate investigation, whereas the pellet was washed once with MeOH:water (2:1), three times with EtOH, and three times with MeOH to remove any free metabolites. Each time, 3 mL of chilled solvent was added, and the pellet was resuspended by vortex mixing, followed by shaking on a VWR multitube vortexer (Model DVX-2500) at 1200 rpm for 2 min, and centrifugation at 3000*g* for 5 min at −5 °C. The supernatants were removed, and the pellets were retained for the next steps.

#### 2.4.3 Detection of Protein-Bound Nitrofurazone Metabolites Using Pseudo-Incurred Samples

##### 2.4.3.1 Protein Substitution Using Mercaptoethanol

For substitution of protein by mercaptoethanol, 500 μL of carbonate buffer (0.05 M, pH 10.5) containing 1 μL of mercaptoethanol (0.2% mercaptoethanol in carbonate buffer) was added to the pellet from the sample prepared in Section 2.4.2. The tube was vortex-mixed vigorously for 15 s to disperse the pellet. The tubes were shaken in a VWR multitube vortexer at 1200 rpm and 25 °C for 10 min. After centrifuging at 15,000 rpm for 2 min, 200 μL of the supernatant was transferred to an Eppendorf tube and acidified to pH ≤ 2 with 10 μL of formic acid. After centrifuging again, the supernatant was transferred to amber vials with inserts for UPLC–HRMS analysis.

### 2.5 UPLC-MS Platforms Used

#### 2.5.1 Identification and Characterization with HRMS

Analysis of the incubation samples was carried out using a Vanquish (Thermo Fisher) UPLC coupled with a hybrid quadrupole-orbitrap HRMS (Exploris 240, Thermo Fisher). A Waters HSST3 column (2.1 × 100 mm, 1.8 μm) held at 30 °C was used. The mobile phases were A: 0.1% formic acid in H_2_O, and B: 0.1% formic acid in 9:1 MeCN: H_2_O. Gradient elution at 0.35 mL/min was performed by holding at 3% B for 1 min, then increasing from 3–10% B over 3 min, 10–70% over 7 min, and finally 70–90% over 0.1 min. The conditions were held at 90% B for 1.9 min before returning to initial conditions over 0.1 min and held for 2.9 min to re-equilibrate.^37^ The injection volume was 5 μL, and the autosampler temperature was held at 5 °C.

##### 2.5.1.1 Full-Scan Data

Full-scan data in the range of *m*/*z* 60–600 was acquired using HRMS with a resolution of 240,000 (FWHM @ *m*/*z* 200) for high mass accuracy and confident identification. The data were obtained in positive-ion mode using heated electrospray ionization (HESI) at 3500 V. The capillary temperature was 325 °C, and the vaporizer was set to 350 °C. The sheath, auxiliary, and sweep gases were set to 50, 10, and 1 (arbitrary units), respectively.

##### 2.5.1.2 MS/MS Data

After suspected nitrofurazone residues were identified from full-scan data, additional injections using full-scan data-dependent MS/MS (FS/ddMS^2^) were performed to acquire high-resolution MS/MS data. An inclusion list with the *m*/*z* values for suspected metabolites, either bound to thiol agents or deconjugated, was used, and MS/MS data were collected at a resolution of 15,000 (FWHM @ *m*/*z* 200) with an isolation window of 1.2 *m*/*z*. The MS/MS spectra were acquired using a normalized collision energy setting of 30%.

#### 2.5.2 MS^3^ Studies for Structure Characterization of Metabolite Conjugates

For detailed characterization of fragmentation pathways, MS^3^ experiments were performed using a Shimadzu UPLC model CBM-20A, coupled to a Q–LIT–MS (SCIEX: Q-Trap 5500), which was used in positive-ion mode for MS^3^ studies with the same UPLC conditions as those given in Section 2.5.1. The MS^3^ scan was acquired with a scan rate of 1000 Da/s. The *m*/*z* values of the conjugated compounds and their product ions were configured as the first and second precursor ions, respectively. The MS parameters were set as follows: declustering potential (DP): 40 V; entrance potential (EP): 10 V; collision energy (CE): 10 eV; excitation energy (AF2): 0.1 eV; collision energy spread (CES): 0. The source/gas parameters were as follows: Curtain gas: 30 psi, collision gas: high, ion spray voltage: 4500 V, temperature: 600 °C, ion source gas 1 (SG1): 50 psi, and ion source gas 2 (SG2): 60 psi. The *m*/*z* range was set from *m*/*z* 60 to the *m*/*z* value of each second precursor ion plus 5.

#### 2.5.3 Quantification of the Metabolite Conjugate

QqQ–MS offers high sensitivity and selectivity for quantification. A Thermo Fisher Vanquish UPLC coupled to a Thermo Fisher TSQ Altis Plus QqQ–MS was operated in Multiple Reaction Monitoring (MRM) mode. The column, mobile phases, flow rate, and injection volume were the same as in Section 2.5.1. To improve peak shape for detection (and avoid broad peaks), a gradient with a shorter run time was used that started at 10% B and held for 0.1 min, then increased from 10% to 90% B over 2.9 min, and from 90 to 95% B over 0.2 min. The conditions were held at 95% B for 1.8 min before returning to initial conditions in 0.2 min and held for 1.8 min to re-equilibrate. MRM data were acquired using positive mode with a HESI spray voltage of 3500 V. The ion transfer tube temperature was 325 °C, and the vaporizer temperature was 350 °C. The sheath, auxiliary, and sweep gases were set to 60, 15, and 1 (arbitrary units), respectively. The optimized collision energies (CEs) are given in Table 1; the RF lens was set at 40 V.

**Table 1:** MRM Transitions Used for the Quantification of the Mercaptoethanol-Metabolite Conjugate^a^

nitrofurazone residue	ESI	precursor ion *m*/*z*	product ion *m*/*z* (optimized CE (eV))
Mercaptoethanol-metabolite conjugate	+	245.1	210(10)/199(12)/184(12)/ 170(8)/167(10)/ 156(15)/124(15)/76(8)
Mercaptoethanol-metabolite-[^13^C,^15^N_2_] conjugate	+	248.1	231(8)/213(10)/202(12)/186(12)/170(8)/158(15)/ 79(8)

a m/*z* 245.1 → 76 was used for quantification in Section 3.4.

When high-field asymmetric waveform ion mobility spectrometry (FAIMS) was used (i.e., UPLC–FAIMS–MRM), the UPLC and MRM conditions remained the same. For FAIMS, standard operating conditions were used, except that the electrode temperatures were set to 80 °C. The compensation voltage (CV) optimization was carried out using the “scan CV” feature in the Tune software (as described previously^38^) and set to CV = −18 V.

### 2.6 Compound Identification

UPLC–HRMS spectra were analyzed with a compound discoverer (CD) workflow reported previously,^37^ and the use of a stable label in the workflow was helpful for identifying nitrofurazone metabolites and conjugates.^39^

## 3 Results and Discussion

### 3.1 *In Vitro* Investigations of Nitrofurazone Using Bovine Liver S9 Fractions

Nitrofurazone metabolism was investigated *in vitro* using bovine liver S9 fractions as described in Section 2.3. The liver S9 fraction is widely used in *in vitro* drug metabolism studies because it enables the simultaneous assessment of phase I and phase II metabolic processes, providing a more comprehensive metabolic profile than microsomes alone.^45^ We selected liver S9 fractions as they are commonly applied for metabolite identification, prediction of *in vivo* metabolic pathways, evaluation of metabolic stability, and assessment of potential drug–drug interactions.^45^

Both free (Section 3.1.1) and bound metabolites (Sections 3.1.2–3.1.4) were examined. UPLC–HRMS spectra were collected (Section 2.5.1) in both positive and negative ion modes and analyzed using Thermo CD 3.3 software. However, only the positive-ion mode results are discussed below, as negative ionization gave similar intensities and did not provide any additional metabolite information.

#### 3.1.1 Free Metabolites in Solution and Formation of Bound Metabolites

The *in vitro* metabolism was performed in 1 and 4 h incubations. The supernatant was analyzed using UPLC–HRMS,^37^ and ion abundance of the protonated nitrofurazone ([M + H]^+^ at *m*/*z* 199.0458, mass error (Δ): −2.0 ppm) remaining in the supernatant is given in Table S1. The dominant nitrofurazone metabolite observed in the supernatant was a cyano-metabolite C_6_H_8_N_4_O_2_^+^ ([M + H]^+^ ion at *m*/*z* 169.0718, Δ: −1.2 ppm). The cyano-metabolite has been previously reported as the major metabolite in catfish,^24^ chicken liver microsomes and S9 fractions,^32^ and *Escherichia coli* cultures.^32^

Both cyano-metabolite-A and/or the reactive intermediate (Scheme 1) possess an electron-withdrawing cyano group and an α,β-unsaturated C–C bond, making them ideal Michael acceptors. Unlike cyano-metabolite-B, this means they can readily react with nucleophilic groups on amino acids or nucleic acids to produce bound metabolites.^36^ After quenching and centrifuging the nitrofurazone-incubated samples (Section 2.3.2), free metabolite was observed in the supernatant as discussed above, whereas the bound metabolite resided in the pellet. Based on a previous study,^8^ pH plays a significant role in the reactions between proteins and nitrofuran metabolites. Vroomen et al. reported that the thiol-bound metabolites were stable at pH < 2. However, the protein-bound metabolites were reported to be stable at a pH range of 7–10, with decreased stability at pH > 11.^8^

#### 3.1.2 Releasing Bound Metabolites and GSH Substitution

Regulatory methods for nitrofurazone use a strong acid to cleave the SEM group from the bound metabolites (Scheme 1). However, our goal was to release the entire metabolite (reactive intermediate and/or cyano-metabolite-A), as this would produce a specific marker for nitrofurazone. Thus, we tested the effect of using thiols on the reversibility of the protein-bound nitrofurazone metabolites. After the supernatant and pellet were separated (and the pellet washed) in the nitrofurazone metabolism samples, a phosphate buffer containing GSH was added to the pellet (Section 2.3.3.1). The basic medium (pH 11) releases the protein-bound metabolites, which can bind to the thiol group in GSH (reconjugation) in a process called substitution. These thiol-metabolite conjugates can arise from the specific reactive intermediate and/or cyano-metabolite-A. Equilibria will be established between any unbound metabolites, the protein-bound metabolites, and the GSH-conjugates based on their reactivities, the pH used, the thiol’s p*K*_a_ values, and the concentration of reagents.

Results from the supernatant were presented in Section 3.1.1. The pellet was washed until no residual cyano-metabolites were detected using the UPLC–HRMS qualitative analysis. The data for the supernatant and rinses are presented in Table S2. The washing steps ensure that any GSH-conjugate detected originates from the protein-bound metabolites. Figure 1 shows the UPLC–HRMS extracted-ion chromatograms of the negative control and the sample for the GSH-conjugate of (A) nonlabeled metabolite ([M + H]^+^ at *m*/*z* 474.1401 ± 5 ppm) and (B) labeled metabolite ([M + H]^+^ at *m*/*z* 477.1374 ± 5 ppm), where the protein-bound metabolite was initially formed from either cyano-metabolite-A or the reactive intermediate (Scheme 1). Based on theoretical *m*/*z* values of the GSH-conjugates, the mass error for the nonlabeled conjugate (*m*/*z* 474.1393) is −1.8 ppm, and the labeled conjugate *(m*/*z* 477.1367) is −2.9 ppm in Figure 1C. The peak splitting (*t*_R_ = 4.61 min and *t*_R_ = 4.67 min) is attributable to the addition of L-GSH to the double bond, creating a new chiral center in the conjugate. Since this addition is not enzyme-catalyzed, it is not likely stereoselective, meaning that the product will likely consist of similar proportions of two diastereoisomers, which often separate using UPLC–HRMS. Thus, seeing a double peak in Figure 1 is consistent with the formation of a GSH-conjugate.

**Figure 1:**
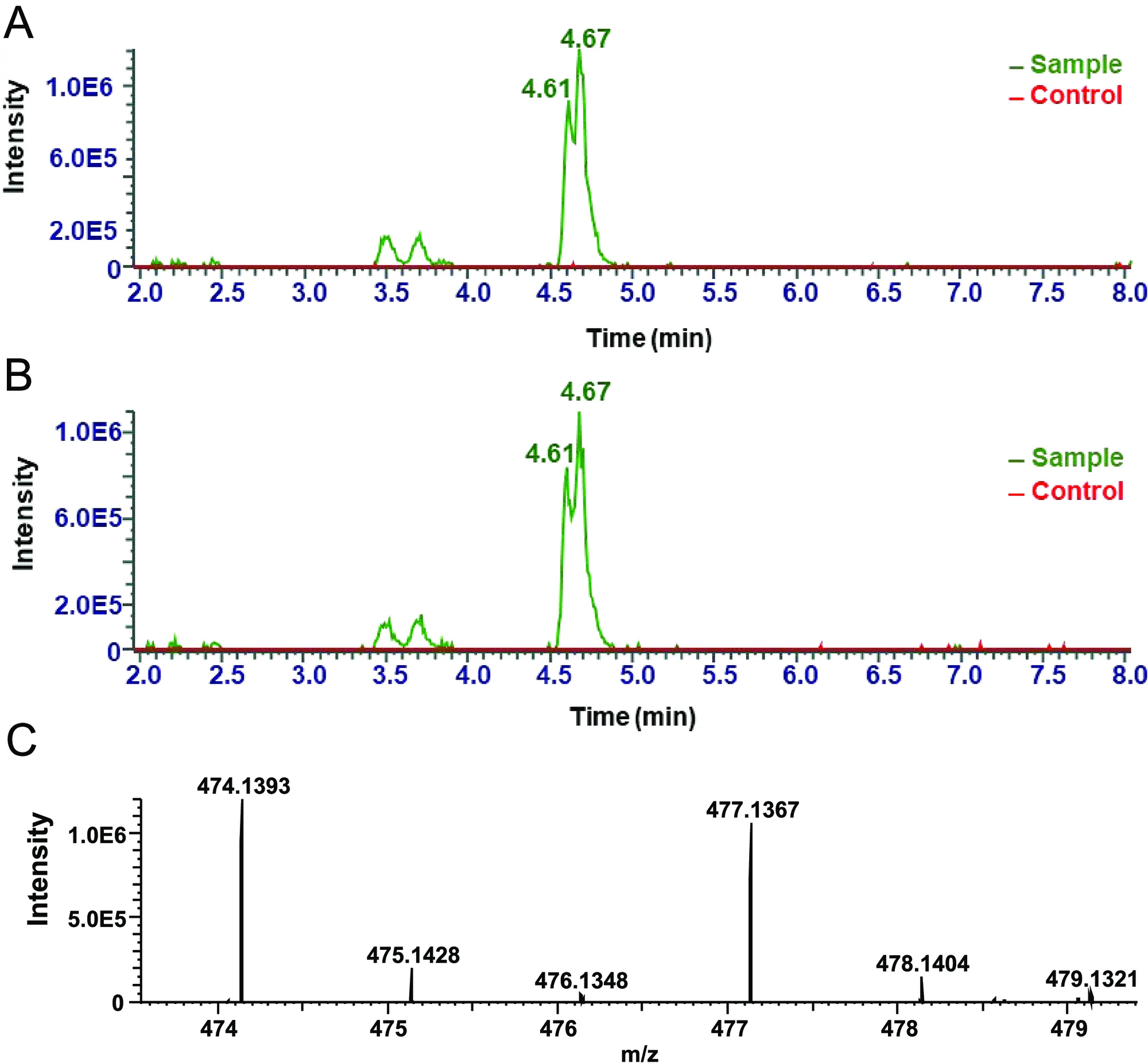
UPLC–HRMS extracted-ion chromatograms for the GSH-conjugate of (A) the metabolite ([M + H]^+^ at *m*/*z* 474.1401 ± 5 ppm, *t*_R_ = 4.61 and 4.67 min) and (B) the labeled metabolite-[^13^C,^15^N_2_] ([M + H]^+^ at *m*/*z* 477.1374 ± 5 ppm, *t*_R_ = 4.61 and 4.67 min). In both (A) and (B), the metabolism sample in green is overlaid with the control shown in red. (C) Mass spectrum at *t*_R_ = 4.67 min shows the presence of the GSH-conjugate of the labeled and nonlabeled metabolite, confirming the replacement of protein with GSH in bound metabolites when using a 1:1 mixture of nonlabeled and isotopically labeled nitrofurazone in the metabolism sample.

Furthermore, a low-level amount of a free cyano-metabolite ([M + H]^+^ ion at *m*/*z* 169.0718; Δ: −1.2 ppm) at *t*_R_ = 4.2 min was observed, which could be evidence of deconjugation occurring for the protein-bound metabolite (Figure S1). Although the reactive intermediate with the exact mass of *m*/*z* 167.0563 was not detected (Scheme 1), this could be due to a rapid reduction after deconjugation (possibly to cyano-metabolite B) and does not exclude conjugation/deconjugation occurring through the reactive intermediate, as a reduction could occur after GSH conjugation to give the same conjugate (Scheme 1). Recall that only cyano-metabolite-A and the reactive intermediate can undergo a Michael addition (and subsequent deconjugation). The mechanism will be explored further in Section 3.3. Table 2 shows the exact mass, chemical formula, and structure of the nitrofurazone used, and the GSH-conjugate produced, along with their isotopically labeled forms.

**Table 2:**
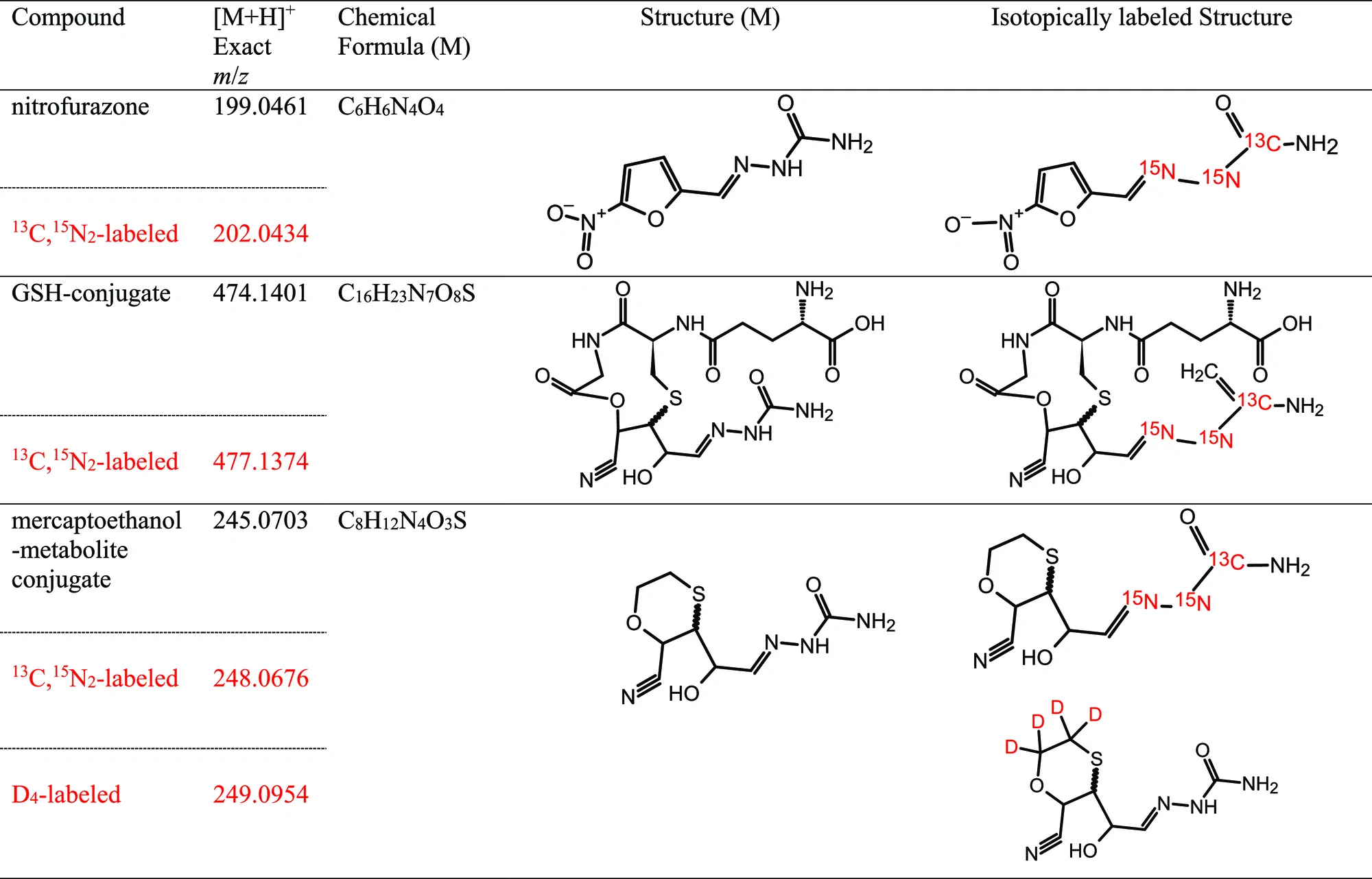
Exact Mass, Chemical Formula, and the Proposed Structure of Thiol-Metabolite Conjugates and Their Related Isotopically Labeled Structures

With UPLC–HRMS, the area of the GSH-conjugate during formation was monitored over time (Table S3), and the optimal reaction time was ∼15 min. Following the deconjugation/substitution process, the GSH-conjugate peak area showed a slow decrease with time (Figure 2). No GSH-conjugate peaks were detected in the sample left at 4 °C overnight, meaning that overnight analysis for a large number of test samples would be challenging due to conjugate degradation. Some degradation appears to occur with the cleavage of γ-glutamyl and SEM from the GSH-conjugate, as a [M + H]^+^ peak is observed at *m*/*z* 320.0908 (C_11_H_18_N_3_O_6_S^+^, Δ: −0.9 ppm).

**Figure 2:**
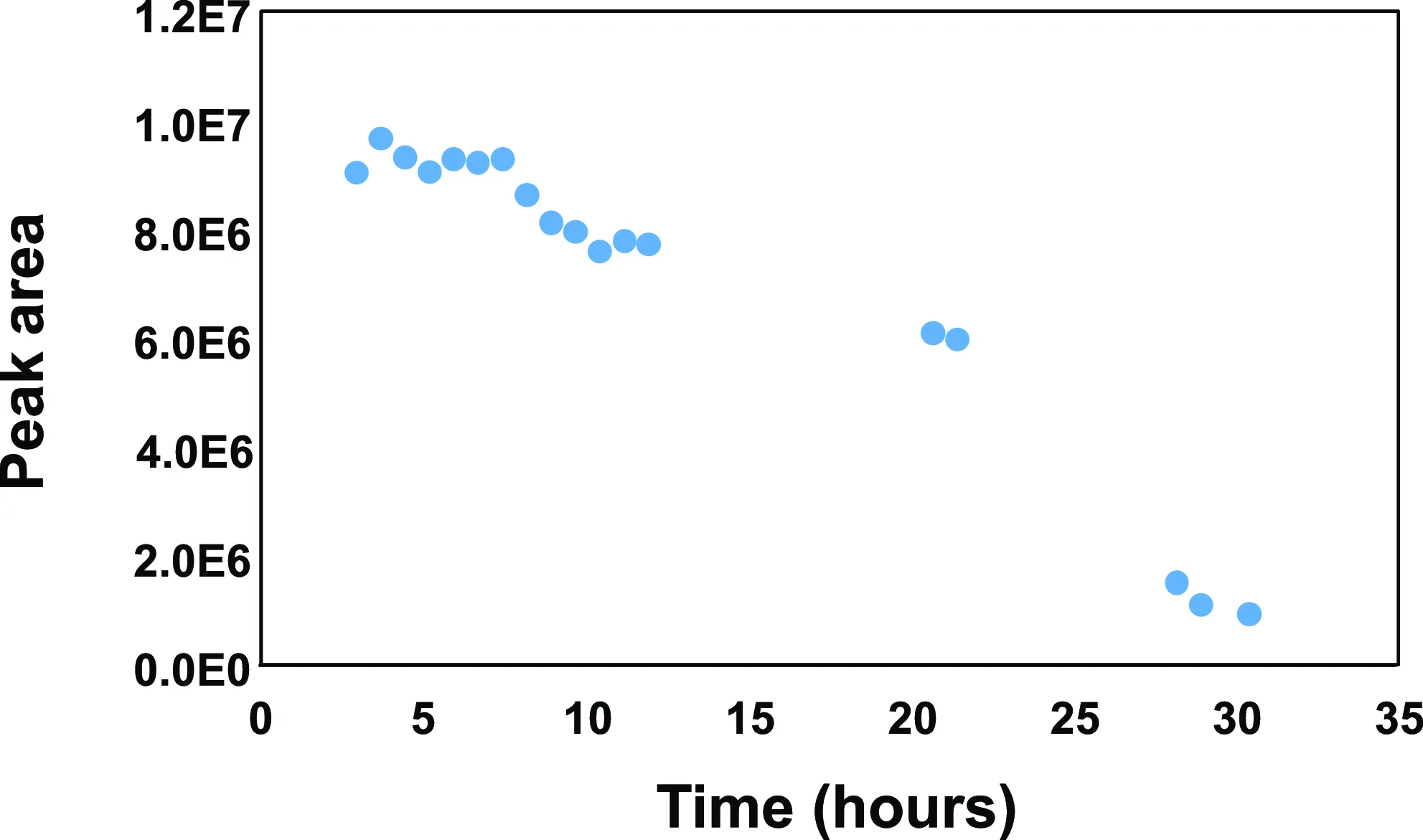
Gradual degradation of the GSH-conjugate. The area of the [M + H]^+^ ion of the GSH metabolite conjugate (at *m*/*z* 474.1401) as a function of time.

#### 3.1.3 Releasing Bound Metabolites and Mercaptoethanol Substitution

A more stable thiol-metabolite conjugate was desired as a reliable marker for nitrofurazone residue detection. Therefore, the deconjugation/substitution process was performed with a non-natural thiol,^36^ mercaptoethanol. Similar to GSH, after an *in vitro* incubation using isotopically labeled and nonlabeled nitrofurazone, a substitution reaction was carried out. A basic buffer containing 0.2% mercaptoethanol was added to the pellet to release protein-bound nitrofurazone metabolites that were subsequently conjugated by mercaptoethanol. The carbonate buffer concentration, pH, mercaptoethanol volume, and reaction time were also examined (Table S4). Figure 3 shows the UPLC–HRMS extracted-ion chromatograms of the negative control and metabolism sample for the mercaptoethanol conjugates of (A) the nonlabeled metabolite ([M + H]^+^ at *m*/*z* 245.0703 ± 5 ppm) and (B) the labeled metabolite ([M + H]^+^ at *m*/*z* 248.0676 ± 5 ppm). Based on theoretical *m*/*z* values of the mercaptoethanol conjugates, the mass error for the nonlabeled conjugate is −0.8 ppm, and the labeled conjugate is 0.0 ppm in Figure 3C. The exact mass, chemical formula, and the proposed structure of the mercaptoethanol-metabolite conjugate, and the related isotopically labeled structures are provided in Table 2. The cyclic structure proposed for conjugates is rationalized in Section 3.3.

**Figure 3:**
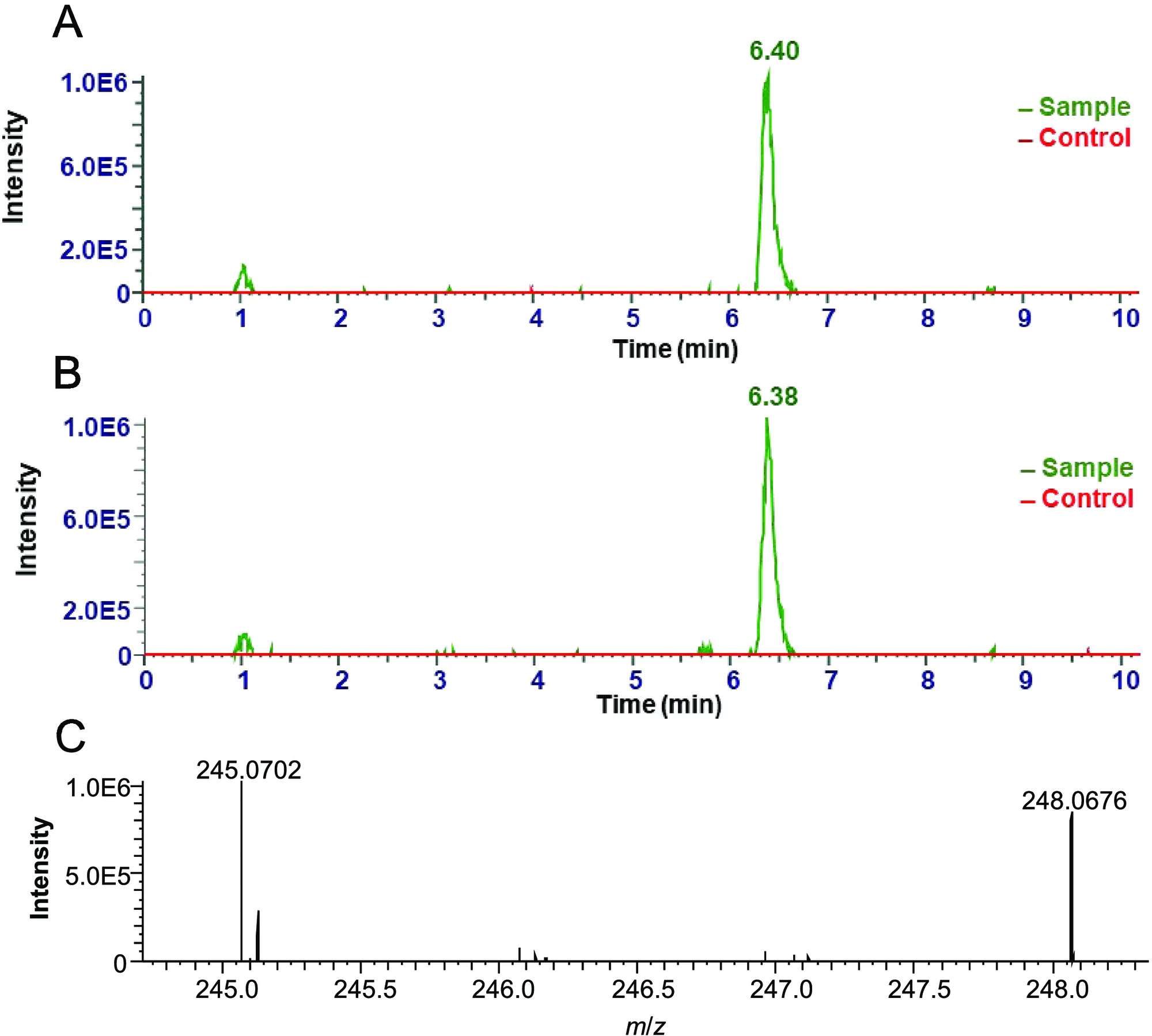
UPLC–HRMS extracted-ion chromatograms for the mercaptoethanol conjugate of (A) the metabolite ([M + H]^+^ at *m*/*z* 245.0703 ± 5 ppm, *t*_R_ = 6.40 min) and (B) the labeled metabolite-[^13^C,^15^N_2_] ([M + H]^+^ at *m*/*z* 248.0676 ± 5 ppm, *t*_R_ = 6.38 min). In both (A) and (B), the metabolism sample in green is overlaid with the control shown in red. (C) The mass spectrum at *t*_R_ = 6.40 min shows the presence of the mercaptoethanol conjugate of the labeled and nonlabeled metabolite, confirming the replacement of protein with mercaptoethanol in bound metabolites when using a 1:1 mixture of nonlabeled and isotopically labeled nitrofurazone in the metabolism sample.

The advantages of working with mercaptoethanol compared with GSH are that it is a liquid at room temperature, and it does not degrade. Additionally, mercaptoethanol is an achiral thiol, which limits the number of isomers. Although its conjugate will be a mixture of enantiomers, it was detected as a single peak (as observed in Figure 3). Furthermore, a furazolidone mercaptoethanol-metabolite conjugate was previously shown to be stable at pH < 2;^8^ therefore, in our work, formic acid was added to the deconjugation supernatant after centrifugation (Section 2.3.3.2). When we tested the acidified mercaptoethanol-metabolite conjugate for stability, it remained stable for at least 6 days (not shown). Lowering the pH after substitution helps to stabilize the conjugate prior to analysis by UPLC–HRMS. Furthermore, some free cyano-metabolite at *t*_R_ = 4.8 min was observed when the carbonate buffer contained high concentrations of mercaptoethanol (e.g., 20%), which indicates an equilibrium between unbound metabolites, the protein-bound metabolites, and the mercaptoethanol conjugates based on their reactivities, the pH used, the thiol’s p*K*_a_ values, and the concentration of reagents. Note that chromatography, including the broad peaks, was adversely affected by the high content of mercaptoethanol (data not shown).

#### 3.1.4 Releasing Bound Metabolites via Deconjugation

The deconjugation of protein-bound metabolites was investigated in basic carbonate buffer and analyzed by UPLC–HRMS. Figure 4 shows the extracted-ion chromatograms for the negative control and metabolism samples for (A) a nonlabeled cyano-metabolite ([M + H]^+^ at *m*/*z* 169.0720 ± 5 ppm) at *t*_R_ = 4.28 min, and (B) a labeled cyano-metabolite ([M + H]^+^ at *m*/*z* 172.0693 ± 5 ppm) at *t*_R_ = 4.27 min. Based on theoretical *m*/*z* values of either cyano-metabolite, the mass error for the nonlabel is −1.8 ppm, and the labeled is −1.2 ppm in Figure 4C. This deconjugation process was examined using different pH values of carbonate buffer (pH 9, 9.5, 10, 10.5, 11) and various incubation times from 2 min to 2 h (data not shown). Although the values across this range were similar, results at pH 9.5 with the carbonate buffer (0.05 M) and an optimized incubation time of 15 min were slightly better and were chosen to release the protein-bound metabolite. Note that the retention times of the free metabolite (cyano-metabolite-B) and the deconjugated cyano-metabolite were not distinguishable.

**Figure 4:**
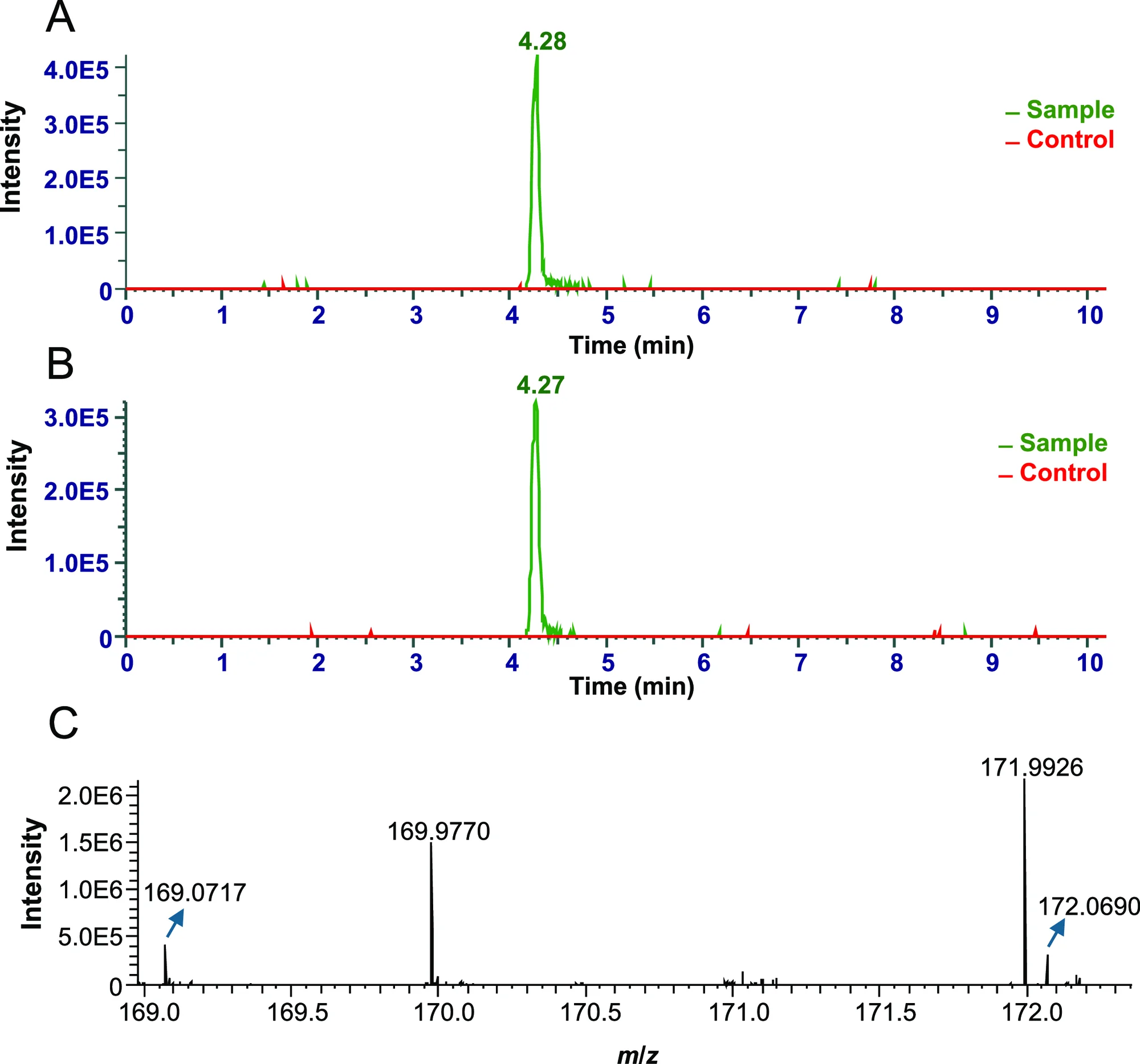
UPLC–HRMS extracted-ion chromatograms of (A) a cyano-metabolite ([M + H]^+^ at *m*/*z* 169.0720 ± 5 ppm, *t*_R_ = 4.28 min) and (B) a labeled cyano-metabolite-[^13^C,^15^N_2_] ([M + H]^+^ at *m*/*z* 172.0693 ± 5 ppm, *t*_R_ = 4.27 min). In both (A) and (B), the metabolism sample in green is overlaid with the control shown in red. (C) The mass spectrum at *t*_R_ = 4.28 min shows a labeled and nonlabeled free cyano-metabolite, confirming the release of the bound metabolite when using a 1:1 mixture of nonlabeled and isotopically labeled nitrofurazone in the metabolism sample.

### 3.2 Investigation of Nitrofurazone Bound Metabolites Using Pseudo-Incurred Samples of Bovine Liver

As deconjugation with only buffer was the simplest method, we proceeded to test the method using pseudo-incurred samples (Section 2.4). A range of basic media using carbonate buffer pH 9–11 was used without any thiol reagent to attempt to release the cyano-metabolite-A (or the reactive intermediate) from protein-bound metabolites produced via pseudo-incurred bovine liver. No detectable cyano-metabolites (or the reactive intermediate) were observed, suggesting that deconjugation without substitution is not a viable approach for pseudo-incurred bovine liver samples. A previous study investigating bound nitrofurazone metabolites in rat liver identified albumin as the primary protein that binds to the metabolite.^40^ The likely reason deconjugation was unsuccessful using only buffer for pseudo-incurred bovine liver samples originates from limitations of S9 fractions, which lack some of the proteins present in liver tissue, including albumin. A Michael addition involving the cysteine thiol in albumin and the α,β-unsaturated cyanide group on the metabolite is a possible reaction site, as these kinds of reactions on cysteine in albumin have been reported as albumin-based drug delivery concepts.^41^ This result highlights that, although S9 *in vitro* incubations are useful in predicting metabolites, they have limitations for testing pseudo-incurred samples.

#### 3.2.1 Mercaptoethanol Substitution in Pseudo-Incurred Samples of Bovine Liver

The same procedure using mercaptoethanol to release bound metabolites *in vitro* was investigated using pseudo-incurred samples of bovine liver. Both isotopically labeled and nonlabeled nitrofurazone were used in the pseudo-incurred bovine liver sample prepared by incubating over 18 h at RT. The carbonate buffer concentration, pH, mercaptoethanol volume, and reaction time were evaluated across a wide range, then narrowed to the conditions shown in Table S5, and the optimized conditions were: 0.05 M carbonate buffer at pH 10.5 containing 0.2% mercaptoethanol and a 10 min reaction time. As was observed *in vitro* (Section 3.1.3), the mercaptoethanol-metabolite conjugate was more stable (for at least 6 days at 5 °C) at acidic pH ≤ 2,^8^ which greatly slows the exchange between the cyano-metabolite-A (and/or reactive intermediate) and either mercaptoethanol or protein conjugates. The samples were analyzed using UPLC–HRMS (Section 2.5.1). Figure 5 shows that both labeled and nonlabeled metabolites were detected as mercaptoethanol conjugates. The UPLC–HRMS extracted-ion chromatograms of the negative control and the pseudo-incurred sample for the mercaptoethanol conjugates of (A) the nonlabeled metabolite ([M + H]^+^ at *m*/*z* 245.0705; Δ: 0.9 ppm) at *t*_R_ = 6.47 min, and (B) the labeled metabolite ([M + H]^+^ at *m*/*z* 248.0679; Δ: 1.2 ppm) at *t*_R_ = 6.45 min with mass errors based on the *m*/*z* values in (C). The presence of the same conjugate was confirmed using pseudo-incurred samples prepared with a short incubation time (1 h) at temperatures of RT or 37 °C (Table S6). During peer review, analysis of the conjugate was also performed in negative ESI mode, as recent studies reported much higher sensitivity (4 times) for cyano-metabolite detection;^24,33^ however, we observed only ∼50% greater sensitivity in negative mode for mercaptoethanol-metabolite conjugate detection (Table S6).

**Figure 5:**
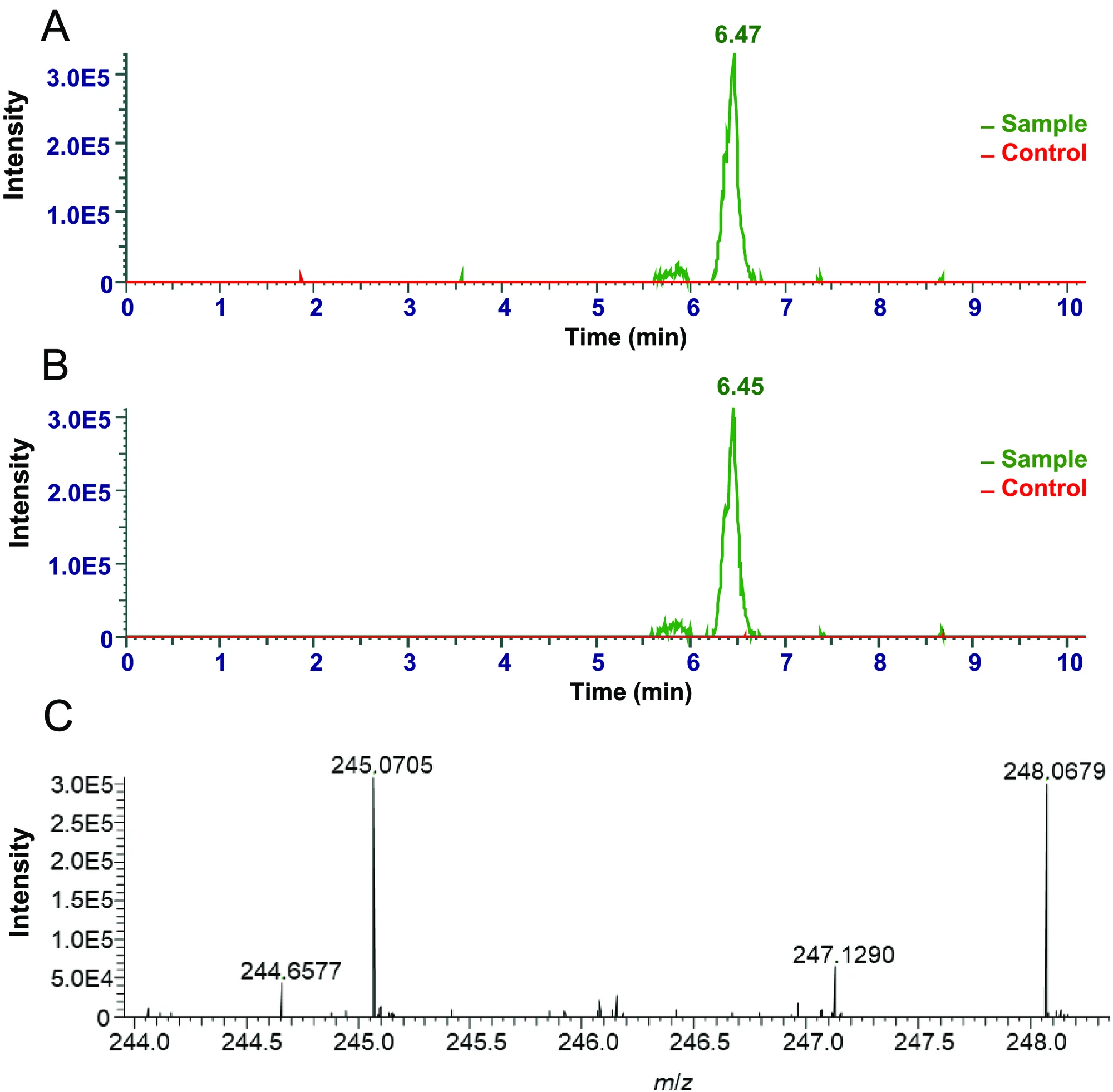
UPLC–HRMS extracted-ion chromatograms for the mercaptoethanol conjugate of (A) the metabolite ([M + H]^+^ at *m*/*z* 245.0703 ± 5 ppm, *t*_R_ = 6.47 min) and (B) the labeled metabolite-[^13^C,^15^N_2_] ([M + H]^+^ at *m*/*z* 248.0676 ± 5 ppm, *t*_R_ = 6.45 min). The pseudo-incurred bovine liver sample in green is overlaid with the control shown in red. (C) The mass spectrum at *t*_R_ = 6.47 min shows the presence of the mercaptoethanol conjugate of the labeled and nonlabeled metabolite, confirming the replacement of protein with mercaptoethanol for the bound metabolite, when using a 1:1 mixture of nonlabeled and isotopically labeled nitrofurazone in the pseudo-incurred bovine liver sample.

### 3.3 Characterizing the Mercaptoethanol Substituted Conjugates

To characterize the mercaptoethanol-metabolite conjugate, bovine liver incurred with a mix of nitrofurazone and isotopically labeled nitrofurazone-[^13^C,^15^N_2_] was used, and the deconjugation/substitution reaction was done using mercaptoethanol. Additionally, bovine liver incurred with nitrofurazone was used, and a mix of mercaptoethanol and isotopically labeled mercaptoethanol-D_4_ was used for the deconjugation/substitution process. Various UPLC–MS-based experiments, including accurate mass measurements, UPLC–MS/MS (Section 2.5.1.2), and UPLC–MS^3^ (Section 2.5.3), were performed to confirm the formation of the mercaptoethanol-metabolite conjugates. Accurate mass measurements showed [M + H]^+^ ions for the mercaptoethanol-metabolite conjugate at *m*/*z* 245.0703, the mercaptoethanol-metabolite-[^13^C,^15^N_2_] conjugate at *m*/*z* 248.0676, and the mercaptoethanol-D_4_ metabolite conjugate at *m*/*z* 249.0954. UPLC–MS/MS spectra for these three conjugates are given in Figures 6A,B, and S2, respectively, with the proposed structure and fragmentation pathway of the nonlabeled conjugate shown in Figure 6C. The detailed MS/MS pattern of the conjugates revealed a dissociation to product ion **P1** via the loss of NH_3_ (Figure 6C). The transition of **P1** to **P3** at *m*/*z* 210.0331 via loss of H_2_O was supported by MS^3^ data (Tables S7 and S8) that were collected using Q–LIT–MS (Section 2.5.3). Another pathway yields the formation of product ion **P2** at *m*/*z* 227.0597 via loss of H_2_O from the **conjugate**. Moreover, **P2** fragments to **P4** at *m*/*z* 199.0284 via loss of the ethylene moiety of the mercaptoethanol, confirmed by label studies (Figure S2 and Table S7). This transition (**P2** to **P4**) in the mercaptoethanol-D_4_ metabolite conjugate fragmentation pattern supports the proposed cyclic structure (Figure S2). A possible acyclic structure with its dissociation pattern is also provided in Figure S3, which indicates that the product ions from the acyclic structure are different from what was observed in the MS/MS spectrum (Figure S2). **P2** converts to **P5** by CHNO cleavage, including C on the SEM portion, confirmed by MS/MS studies on the labeled conjugates (Table S7). The product ion **P7** at *m*/*z* 156.0226 forms by cleavage of the ethylene moiety of **P5**, confirmed by MS^3^ and label studies. In a different pathway, **P5** loses C_2_H_4_S and produces product ion **P8** at *m*/*z* 124.0505. The product ion **P6** at *m*/*z* 170.0270 is formed via loss of SEM, and the product ion **P9** is the SEM ion via loss of neutral **P6**, as proved by label studies.

**Figure 6:**
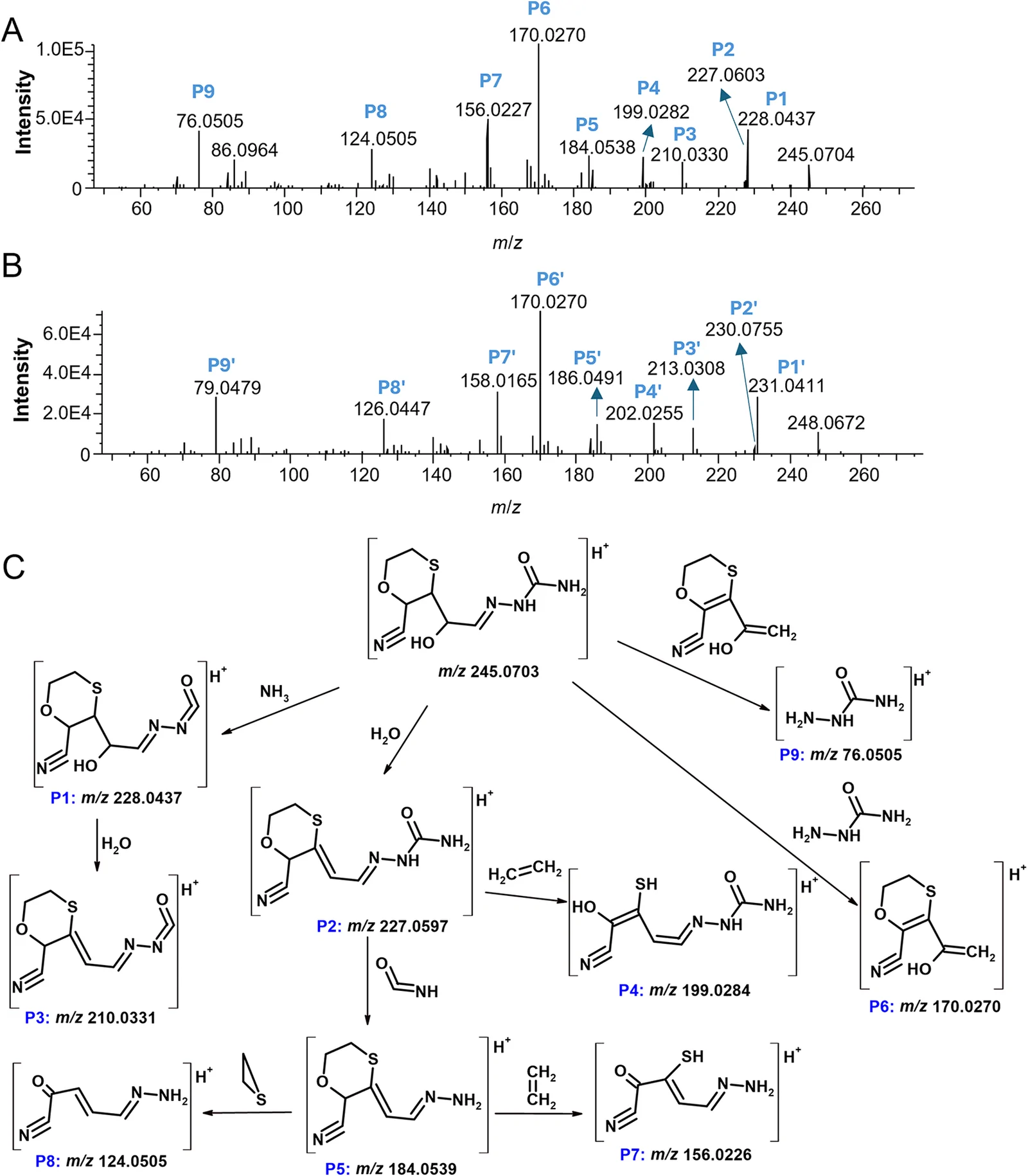
MS/MS spectra of mercaptoethanol conjugates of (A) nonlabeled metabolite ([M + H]^+^ at *m*/*z* 245.0704; Δ: 0.5 ppm) and (B) labeled metabolite-[^13^C,^15^N_2_] ([M + H]^+^ at *m*/*z* 248.0672; Δ: −1.6 ppm) and *t*_R_ = 6.4 min. (C) The proposed MS/MS dissociation behavior for the nonlabeled conjugate.

The structures proposed for **P2**, **P3**, and **P5** are consistent with the product ions of the mercaptoethanol-D_4_ metabolite conjugate, which confirms that all of the deuteriums remain in the product ions (Figure S2). This, along with the two deuteriums on **P4**, strongly supports the proposed structure (recall cyano-metabolite-B is not a Michael acceptor). This observation also supports our proposed structure for thiol conjugation to be on the Cβ of the cyano group. Also, these product ions would seem to support thiol conjugation with the cyano-metabolite-A of nitrofurazone rather than conjugation with the reactive intermediate, unless the conjugate initially formed with the reactive intermediate undergoes subsequent reduction (Scheme 1). There is some evidence for a thiol-metabolite conjugate intermediate, as very small peaks for *m*/*z* 243.0522 and *m*/*z* 246.0521 were observed at *t*_R_ = 7.4 min (Figure S4), compared to the thiol-metabolite conjugate at *t*_R_ = 6.4 min. The *t*_R_ values are also consistent with the −OH conjugate being more polar than the intermediate.

### 3.4 MRM Studies Using the Mercaptoethanol Conjugate as a Specific Marker

The prepared pseudo-incurred liver samples (Section 2.4) were quantified for SEM resulting from protein-bound metabolites of nitrofurazone using an established regulatory method (Supporting Method S1). For bovine liver samples incurred with 0.05–0.4 μg/g nitrofurazone, the results from the regulatory method are shown in Table S9. An MRM analysis of the same pseudo-incurred tissues using the mercaptoethanol-metabolite conjugate was performed with a QqQ MS (Section 2.5.2). The purpose was to assess the detection limit (LOD) of the proposed substitution method. Figure 7A shows the quantitative traces for the mercaptoethanol-metabolite conjugate (*m*/*z* 245.1 → 76.0) for the four pseudo-incurred nitrofurazone samples (blank, 0.05, 0.2, and 0.4 μg/g). The regulatory hydrolysis method detected 3.5 ng/g of SEM for a nominal 0.05 mg/kg nitrofurazone-spiked (i.e., pseudo-incurred) liver sample (Table S9), which would be the LOD of the novel substitution method with UPLC–MRM since the S/N value is ∼3 in Figure 7A. However, UPLC–FAIMS–MRM was used to further improve detection since FAIMS separates ions in the gas phase based on differences in mobility,^42^ and has been used previously to improve S/N in veterinary drug residue detection.^38,43^ Figure 7B shows that the FAIMS essentially eliminates the chemical background noise, giving a much higher S/N of ∼85 for the same nominal 0.05 mg/kg nitrofurazone-spiked (i.e., pseudo-incurred) liver sample. Thus, using UPLC–FAIMS–MRM, the desired European Union minimum performance level of 0.5 ng/g SEM would be achieved.

**Figure 7:**
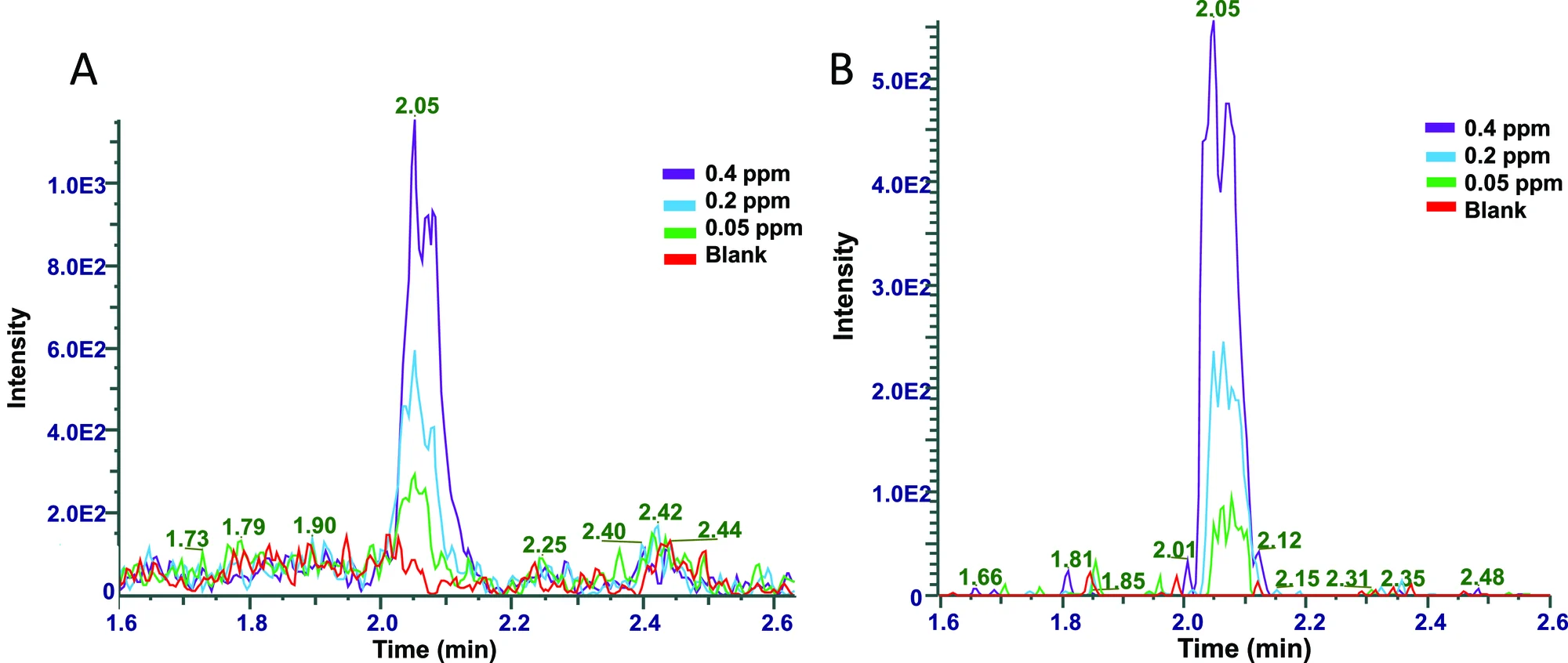
UPLC–MRM chromatograms for the quantitative transition *m*/*z* 245.1 → 76.0 for the mercaptoethanol-metabolite conjugate using 0.0 (blank), 0.05, 0.2, and 0.4 mg/kg pseudo-incurred liver tissue. Unsmoothed data of (A) without FAIMS and (B) with FAIMS at CV = −18 V.

Metabolites of nitrofurazone have been synthesized previously,^44^ and standards will also be needed to do absolute quantification of the amount of the conjugate. For this work, the amount of the mercaptoethanol-metabolite conjugate was estimated using the regulatory method. For this estimate, both the remaining pellet (after the substitution experiment) and a sample of pseudo-incurred tissue were tested. The results showed that the substitution procedure recovered approximately 10% of the protein-bound metabolite. Since the hydrolysis procedure will detect all metabolites containing SEM, one possibility is that other unidentified metabolites could contribute to the hydrolysis. However, the use of the labeled and nonlabeled nitrofurazone eliminates any significant contribution of other metabolites using substitution, as the data analysis software readily finds *m*/*z* differences between the label and nonlabel (Δ*m*/*z* 2.9972) that would be indicative of any other SEM metabolites. Since this is a complex biological system, another possibility is that other binding chemistry is playing a role in the low recovery. However, we believe, at least in part, the low recovery is due to a nonoptimized procedure, and we are currently investigating additional options to improve recovery rates that would further improve LOD values in future studies.

### 3.5 Implications and Future Work

The use of thiol reagents to produce substitution reactions leading to the formation of a mercaptoethanol-metabolite conjugate is a major breakthrough for detecting residues of nitrofurazone. As a specific marker, this conjugate provides conclusive evidence for the use of nitrofurazone, which cannot be obtained from SEM detection using the hydrolysis method. Structural studies support substitution via Michael addition with either cyano-metabolite-A and/or the reactive intermediate (cyano-metabolite-B cannot undergo Michael addition). However, the inability to definitively prove cyano-metabolite-A, combined with the evidence for the conjugate intermediate in the pathway for the reactive intermediate (Figure S4), suggests the conjugate formation occurs through the reactive intermediate (Scheme 1). We agree with previous studies that the free metabolite (i.e., unbound) is cyano-metabolite-B.

When compared with the hydrolysis method, the substitution method is much faster and would be valuable for the analysis of nitrofurans that lack an imine group (e.g., nifurpirinol and nifurstyrenate) and do not yield suitable markers with hydrolysis. Although UPLC-FAIMS-MRM analysis meets the European Union minimum performance level, we are nonetheless currently investigating additional ways to further improve the recovery of the metabolite. Future work will include synthesizing standards and validation of the method. In addition, the use of this method will be extended to aquatic species, in which the use of nitrofurazone is also an issue. The substitution methodology could also be used to investigate other protein-bound residues (e.g., florfenicol-bound metabolites) that suffer from sensitivity or selectivity issues.

## Supplementary Material



## Notes

UPLC–HRMS extracted-ion chromatograms for the cyano-metabolite confirming the release of the bound metabolite (Figure S1); MS/MS spectrum and proposed dissociation behavior of the mercaptoethanol-D_4_ metabolite conjugate (Figure S2); proposed acyclic structure and dissociation behavior of the mercaptoethanol-D_4_ metabolite conjugate (Figure S3); UPLC–HRMS extracted-ion chromatograms showing evidence of a thiol-metabolite conjugate intermediate (Figure S4); monitoring the ion intensity of the nitrofurazone [M + H]^+^ ion remaining in the supernatant (Table S1); monitoring the supernatant and rinses of the pellet in the *in vitro* metabolized sample to ensure free cyano-metabolites are eliminated (Table S2); optimization of incubation time for the substitution of protein by the L-GSH group in the phosphate buffer (0.1 M, pH 11) (Table S3); effect of pH and time for the substitution reaction of the protein-bound metabolite of nitrofurazone with mercaptoethanol in bovine liver S9 fractions (Table S4); effect of pH and time for the substitution of the protein-bound metabolite of nitrofurazone with mercaptoethanol in pseudo-incurred bovine liver (Table S5); effect of temperature on the mercaptoethanol metabolite conjugate peak area when preparing the pseudo-incurred sample (Table S6); theoretical and experimental *m*/*z* values, mass error, and formula for the product ions of the mercaptoethanol-metabolite conjugate (Table S7); MS^3^ analysis of product ions of the mercaptoethanol-metabolite conjugate (Table S8); quantification of SEM in pseudo-incurred nitrofurazone liver samples using a regulatory method (Table S9); and regulatory method for protein-bound metabolites of nitrofurans by LC–MS/MS (Method S1) (PDF)

## List of Abbreviations

NRs: nitrofuran residues
SEM: semicarbazide
UPLC–HRMS: ultraperformance liquid chromatography–high-resolution mass spectrometry
HESI: heated electrospray ionization
UPLC–MS/MS: ultraperformance liquid chromatography–tandem mass spectrometry
Q–LIT: quadrupole linear ion trap
QqQ: triple quadrupole
MRM: Multiple Reaction Monitoring
CD: compound discoverer
GSH: glutathione
GSH-conjugate: glutathione metabolite conjugate
FAIMS: high-field asymmetric waveform ion mobility spectrometry
CV: compensation voltage.
