# Supplementary material for: A Novel Analytical Strategy to Detect Specific Markers of Nitrofurazone from Protein-Bound Metabolites in Food Animals
Source: J Agric Food Chem. 2026 Sep 18;74(38):30296–308. doi: 10.1021/acs.jafc.6c04435 (PMC13632585; doi:10.1021/acs.jafc.6c04435)
Supplement: Supplementary file 1 [file jf6c04435_si_001.pdf]

Supporting Information

A novel analytical strategy to detect specific markers of nitrofurazone from protein-bound metabolites in food animals

Sedigheh Barzegar[a,b], Bryn O. Shurmer[b], Christopher O. Miles[c,d], Anas El-Aneed[a], Randy W. Purves*[a,b]

[a]College of Pharmacy and Nutrition, University of Saskatchewan, Saskatoon, SK, Canada S7N 5E5

[b]Centre for Veterinary Drug Residues, Canadian Food Inspection Agency, Saskatoon, SK, Canada S7N 2R3

[c]Biotoxin Metrology, National Research Council, 1411 Oxford Street, Halifax, NS, Canada. B3H 3Z1

[d]Norwegian Veterinary Institute, Elizabeth Stephansens vei 1, 1433 Ås, Norway

*Corresponding author

Address reprint requests to

Randy W. Purves

Centre for Veterinary Drug Residues, Canadian Food Inspection Agency, Saskatoon, SK, Canada S7N 2R3

Tel: +1 306-385-7843
Email address: randy.purves@usask.ca or randy.purves@inspection.gc.ca

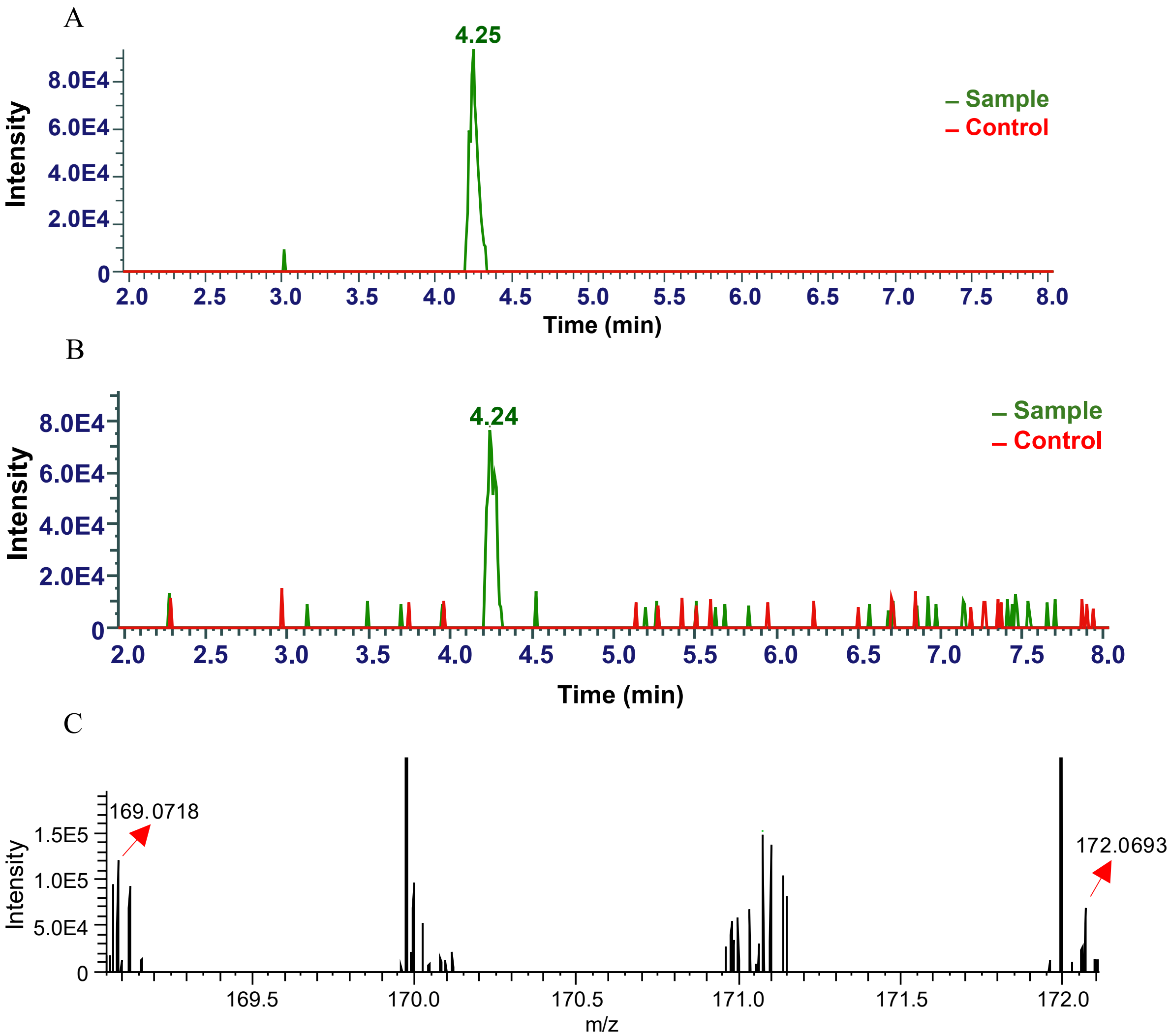

Figure S1. UPLC–HRMS extracted-ion chromatograms for (A) the $[M+H]^+$ ion of the cyano-metabolite at m/z 169.0720 ± 5 ppm (t_R = 4.25 min), and (B) the $[M+H]^+$ ion of the labeled cyano-metabolite-[^{13}C,$^{15}N_2$] at m/z 172.0693 ± 5 ppm (t_R = 4.24 min). In both (A) and (B), the metabolism sample in green is overlayed with the control shown in red. (C) The mass spectrum at t_R = 4.25 min shows the presence of the labeled and nonlabeled free cyano-metabolite at low (near background) levels, confirming the release of the bound metabolite, when using a 1:1 mixture of nonlabeled and isotopically labeled nitrofurazone in the metabolism sample.

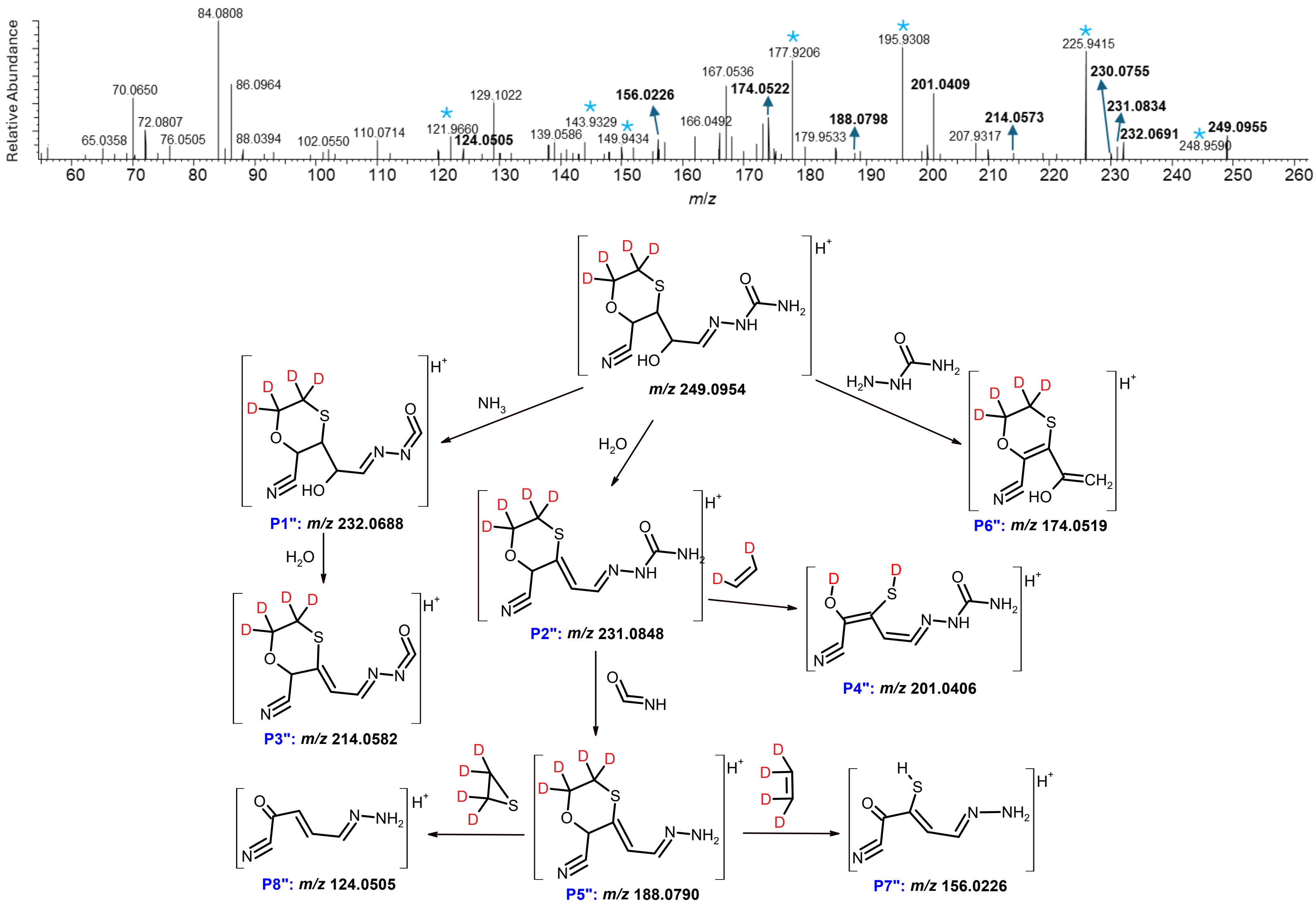

Figure S2. The MS/MS spectrum and proposed dissociation behavior of the mercaptoethanol-D_4 metabolite conjugate (Scheme 1) ($[M+H]^+$ ion at *m/z* 249.0937) to confirm the dissociation pattern of the mercaptoethanol-metabolite conjugate at *m/z* 245.0703. *Contributions from unresolved matrix in the isolation window.

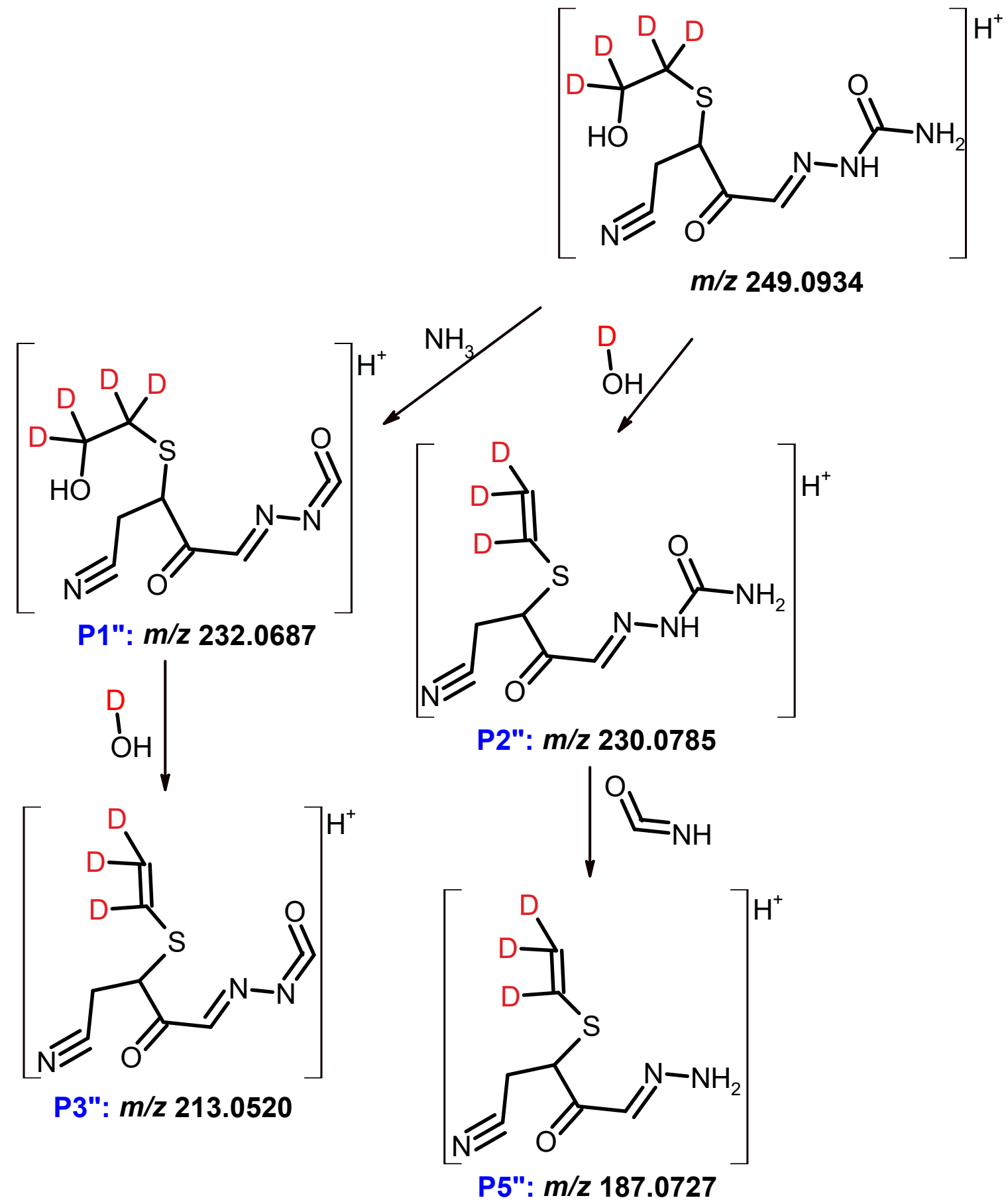

Figure S3. The proposed acyclic structure and dissociation behavior of the mercaptoethanol-D_4 metabolite conjugate. The product ions at *m*/*z* 213.0520 and *m*/*z* 187.0727 are not observed in the MS/MS spectra shown in Figure S2 and the product ion at *m*/*z* 230.0785 does not match with the observed *m*/*z* 230.0755 (Δ: 13.0 ppm) supporting the cyclic structure proposed for the mercaptoethanol-metabolite conjugate (Figure S2).

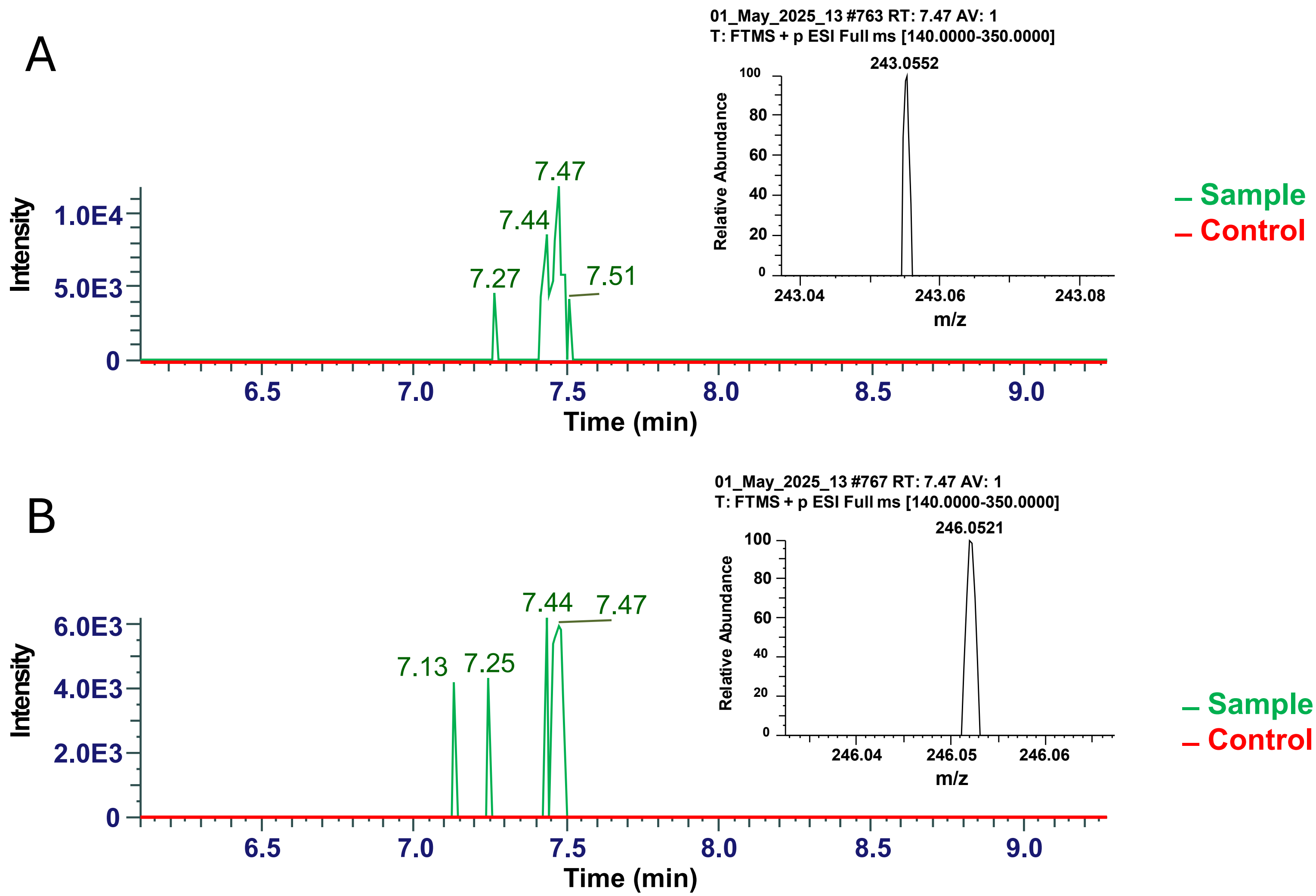

Figure S4. UPLC–HRMS extracted-ion chromatograms for (A) an $[M+H]^+$ ion at m/z 243.0522 ± 5 ppm, and (B) a labeled $[M+H]^+$ ion at m/z 246.0521 ± 5 ppm. In both (A) and (B), the metabolism sample in green is overlayed with the control shown in red. The indexed mass spectrum at t_R = 7.47 min shows evidence of the thiol-metabolite conjugate intermediate (Scheme 1) which then undergoes reduction and converts to the thiol metabolite conjugate.

Table S1. Monitoring the ion intensity of the nitrofurazone $[M+H]^+$ ion at *m/z* 199.0462 ± 5 ppm, remaining in the supernatant of the *in vitro* metabolized sample.

NTF + labeled		Detection of nitrofurazone $[M+H]^+$ ion (*m/z* 199.0458, Δ: −2.0 ppm)	
		t_R (min)	intensity
1 hr. incubation in Primacyt bovine S9, 200 µL of the solution + 300 µL water	Supernatant	7.7	1.26×10^8
1 hr. incubation in Primacyt bovine S9, 200 µL of the solution evaporated and reconstituted by 500 µL methanol: water (5:95)	Supernatant	7.7	2.63×10^8
4 hr. incubation in Primacyt bovine S9, 200 µL of the solution + 300 µL water	Supernatant	7.7	0.98×10^8
4 hr. incubation in Primacyt bovine S9, 200 µL of the solution evaporated and reconstituted by 500 µL methanol: water (5:95)	Supernatant	7.7	1.84×10^8

Table S2. Monitoring the supernatant and rinses of the pellet in the *in vitro* metabolized sample to ensure free cyano-metabolites are eliminated.

NTF + labeled		Intensity of the free cyano-metabolite $[M+H]^+$ ion, *m/z* 169.0718 (Δ: −1.2 ppm)	
		t_R (min)	intensity
1 hr. incubation in Primacyt bovine S9, 200 µL of the solution + 300 µL water	Supernatant	4.3	1.38×10^6
	EtOH 1[a]	4.3	6.13×10^4
	EtOH 2[b]	4.3	9.45×10^3
	EtOH 3[c]	-	0
	MeOH 1[d]	4.2	6.28×10^3
	MeOH 2[e]	-	0
	MeOH 3[f]	-	0
1 hr. incubation in Primacyt bovine S9, 200 µL of the solution evaporated and reconstituted by 500 µL methanol: water (5:95)	Supernatant	4.3	3.70×10^6
	EtOH 1	4.3	3.44×10^5
	EtOH 2	4.3	6.0×10^4
	EtOH 3	4.3	6.40×10^3
	MeOH 1	4.3	9.49×10^3
	MeOH 2	-	0
	MeOH 3	-	0
4 hr. incubation in Primacyt bovine S9, 200 µL of the solution + 300 µL water	Supernatant	4.3	5.69×10^6
	EtOH 1	4.3	2.17×10^5
	EtOH 2	4.3	3.02×10^4
	EtOH 3	4.3	8.78×10^3
	MeOH 1	-	0
	MeOH 2	-	0
	MeOH 3	-	0
4 hr. incubation in Primacyt bovine S9, 200 µL of the solution evaporated and reconstituted by 500 µL methanol: water (5:95)	Supernatant	4.3	8.91×10^6
	EtOH 1	4.3	1.15×10^6
	EtOH 2	4.3	1.04×10^5
	EtOH 3	4.3	1.71×10^4
	MeOH 1	4.3	1.05×10^4
	MeOH 2	-	0
	MeOH 3	-	0

[a] EtOH 1: First washing step using chilled ethanol.
[b] EtOH 2: Second washing step using chilled ethanol.
[c] EtOH 3: Third washing step using chilled ethanol.
[d] MeOH 1: First washing step using chilled methanol.
[e] MeOH 2: Second washing step using chilled methanol.
[f] MeOH 3: Third washing step using chilled methanol.

Table S3. The optimization of incubation time for the substitution of protein by the L-GSH group in the phosphate buffer (0.1 M, pH 11).

		Peak area of the glutathione-metabolite conjugate ion, *m/z* 474.1393 (Δ: −1.8 ppm), t_R = 4.6 min		
	Time (min)	Negative control	Sample1	Sample2
Bovine liver S9 fraction + ($MgCl_2$ & G6P & NADPH)	15	-	1.51×10^7	1.31×10^7
	60	-	9.57×10^6	1.08×10^7
	120	-	9.19×10^6	2.54×10^6
	180	-	9.39×10^5	1.06×10^7
	overnight	-	-	-

Table S4. The effect of pH and time for the substitution reaction of the protein-bound metabolite of nitrofurazone (metabolized for 1 h in bovine liver S9 fractions). Substitution used 0.2% mercaptoethanol in carbonate buffer (formic acid (FA) was added for stability).

Carbonate buffer (0.05 M)	Time of reaction (min)	Peak area of the mercaptoethanol-metabolite conjugate ion, *m/z* 245.0702 (Δ: −0.8 ppm), t_R = 6.4 min		
pH		Negative control	sample	Sample + FA
9.5	2	-	6.0×10^6	6.1×10^6
9.5	10	-	6.6×10^6	8.4×10^6
10.5	2	-	1.1×10^6	2.7×10^6
10.5	10	-	2.7×10^6	4.0×10^6

Table S5. The effect of pH and time for the substitution of the protein-bound metabolite of nitrofurazone (pseudo-incurred bovine liver). Substitution used 0.2% mercaptoethanol in carbonate buffer (formic acid (FA) was added for stability).

Carbonate buffer (0.05 M)	Time of reaction (min)	Peak area of the mercaptoethanol-metabolite conjugate ion, *m/z* 245.0702 (Δ: −0.8 ppm), t_R = 6.4 min		
pH		Negative control	sample	Sample + FA
9.5	2	-	8.4×10^5	1.3×10^6
9.5	10	-	2.1×10^6	1.9×10^6
10.5	2	-	2.8×10^6	2.9×10^6
10.5	10	-	3.4×10^6	3.0×10^6

Table S6. The effect of temperature on the mercaptoethanol metabolite conjugate peak area when preparing the pseudo-incurred sample (2 μg/g of each [^{13}C,$^{15}N_2$]-labeled and nonlabeled nitrofurazone) using a 1 hour incubation. Protein substitution used 0.2% mercaptoethanol in (0.05 M, pH 10.5) carbonate buffer incubated for 10 min (formic acid (FA) was added for stability).

			Peak area of the nonlabeled and [^{13}C,$^{15}N_2$]-labeled mercaptoethanol-metabolite conjugate ions		
ESI mode	Temperature of incubation	t_R (min)	Negative control	Exact mass *m/z* 245.0702	Exact mass *m/z* 248.0679
Positive	37 °C	6.4	-	1.2×10^6	1.1×10^6
Positive	RT*	6.4	-	1.0×10^6	1.0×10^6
			Peak area of the nonlabeled and [^{13}C,$^{15}N_2$]-labeled mercaptoethanol-metabolite conjugate ions		
			Negative control	Exact mass *m/z* 243.0522	Exact mass *m/z* 246.0521
Negative	37 °C	7.4	-	2.0×10^6	2.0×10^6
Negative	RT*	7.4	-	1.4×10^6	1.5×10^6

* RT: Room Temperature

Table S7. The theoretical and experimental *m*/*z* values, mass error, and formula for the product ions of the mercaptoethanol-metabolite conjugate. Additionally, experimental *m*/*z* values for the mercaptoethanol conjugate of metabolite-[^{13}C,$^{15}N_2$] and the mercaptoethanol-D_4 metabolite conjugate and all of their product ions are provided.

Compound	Ion ID	Theoretical *m*/*z*	Experimental *m*/*z*	Formula	Mass error (ppm)	metabolite-[^{13}C,$^{15}N_2$] experimental *m*/*z*	mercaptoethanol-D_4 experimental *m*/*z*
mercaptoethanol metabolite conjugate		245.0703	245.0704	$C_8H_{13}N_4O_3S^+$	0.5	248.0672	249.0934
product ions	P1	228.0437	228.0437	$C_8H_{10}N_3O_3S^+$	−0.2	P1′: 231.0411	P1″: 232.0687
	P2	227.0597	227.0603	$C_8H_{11}N_4O_2S^+$	2.5	P2′: 230.0755	P2″: 231.0847
	P3	210.0331	210.0330	$C_8H_8N_3O_2S^+$	−0.8	P3′: 213.0308	P3″: 214.0586
	P4	199.0284	199.0282	$C_6H_7N_4O_2S^+$	−1.1	P4′: 202.0255	P4″: 201.0406
	P5	184.0539	184.0538	$C_7H_{10}N_3OS^+$	−0.6	P5′: 186.0491	P5″: 188.0783
	P6	170.0270	170.0270	$C_7H_8NO_2S^+$	−0.2	P6′: 170.0270	P6″: 174.0519
	P7	156.0226	156.0227	$C_5H_6N_3OS^+$	0.6	P7′: 158.0165	P7″: 156.0222
	P8	124.0505	124.0505	$C_5H_6N_3O^+$	0	P8′: 126.0447	P8″: 124.0505

Table S8. MS^3 analysis of product ions of the mercaptoethanol metabolite conjugate.

Precursor ion *m/z*	Product ion	First generation product ions *m/z* (MS/MS)	Second generation product ions *m/z* (MS^3)
245.0703	P1	228.0437	211.1, 210.1, 200.1, 185.1, 182.1, 172.1, 168.0, 144.1, 112.1, 89.0
	P2	227.0603	199.0, 184.0, 172.0, 156.0, 124.1, 96.1
	P3	210.0330	182.0, 168.1, 150.0
	P4	199.0282	172.0, 156.0, 148.9, 144.4
	P5	184.0538	167.1, 157.0, 155.9, 124.1, 111.2, 96.1
	P6	170.0270	142.0, 114.1
	P7	156.0227	129.1, 126.2, 111.0, 109.0, 100.9, 83.9, 74.1

Table S9. Quantification of SEM in pseudo-incurred nitrofurazone liver samples using a regulatory method (Supporting Method S1).

Nitrofurazone Spike (ppm)	SEM Concentration (ppb)	Mean SEM Concentration (ppb)
0.05	3.7	3.5
0.05	3.3	
0.1	8.0	8.0
0.1	8.1	
0.2	14.5	14.8
0.2	15.2	
0.4	29.4	31.3
0.4	33.2	

Note: In producing these spikes, it was determined that approximately 20% of the nitrofurazone binds to the proteins. With the stoichiometric conversion from parent drug to SEM, a 0.05 ppm nominal nitrofurazone spike contains 3.5 ppb of bound SEM.

Method S1

Regulatory method for protein-bound metabolites of nitrofurans by LC–MS/MS.

Sample preparation

A diagnostic regulatory method based on hydrolysis followed by SEM derivatization was used. The sample preparation method included the removal of extractable metabolites in which 2.0 g of pseudo-incurred tissue were weighed and washed once with MeOH: water (2:1), three times with EtOH, and three times with MeOH to remove any free metabolites. Each time, 6 mL of chilled solvent was added, and the pellet was resuspended by sonication, and centrifuged at 3,000 g for 10 min at −5 °C. The supernatants were removed, and the pellets were dried under N_2. Then, the overnight digestion in the presence of hydrochloric acid and 2-nitrobenzaldehyde at 37 °C was performed, which has been described elsewhere [28]. After neutralizing and extraction by 6 mL ethyl acetate (3 times), the dried extract was dissolved in water [28]. In the next step, 810 µL water was added and extracted with 6 mL hexane (3 times). The mixture was centrifuged each time at 1000 rpm for 5 min at −5 °C and the hexane layer was discarded. Any remaining traces of hexane were evaporated under a gentle stream of nitrogen, and 90 µL of 1.0% acetic acid in acetonitrile was added to the aqueous solution and vortex mixed. The final solution was filtered through a 0.2 µm PTFE filter into an autosampler vial.

UPLC–MRM analysis

Analysis was carried out using a Acquity I class (Waters) UPLC coupled to a Waters Xevo TQ-S micro triple-quadrupole mass spectrometer for LC–MRM studies. An HSS PFP column (2.1 × 30 mm, 1.8 µm) held at 45°C was used. The mobile phases were A: acetonitrile and B: 1% acetic acid in H_2O, with the gradient elution at various flow rates showed in the Table A1.

Table A1. Mobile phase gradient (A: acetonitrile and B: 1% acetic acid in H_2O).

Time (min)	Flow rate (mL/min)	%B
initial	0.999	8.0
0.10	0.999	8.0
0.48	0.999	35.0
0.55	0.410	35.0
1.42	0.425	60.0
1.45	0.700	98.0
2.00	0.800	98.0
2.05	0.750	8.0
2.15	0.999	8.0
3.00	0.999	8.0

The injection volume was 5 μL and the autosampler temperature was held at 5 °C. MRM data was acquired using positive mode with a Unispray with impactor and Cone voltages of 0.85 kV and 29 V, respectively. The desolvation temperature was 600 °C, source temperature was 150 °C, and the desolvation and cone gas flow were 1200 and 75 L/hr, respectively. MRM transitions of *m*/*z* 209.00 → 133.96 (CE: 14), *m*/*z* 149.00 (CE: 14), *m*/*z* 166.10 (CE: 9), *m*/*z* 192.10 (CE: 9) were used for quantification.
